# CRISPR/sgRNA-directed synergistic activation mediator (SAM) as a therapeutic tool for Parkinson´s disease

**DOI:** 10.1038/s41434-023-00414-0

**Published:** 2023-08-04

**Authors:** Luis Fernando Narváez-Pérez, Francisco Paz-Bermúdez, José Arturo Avalos-Fuentes, Aurelio Campos-Romo, Benjamín Florán-Garduño, José Segovia

**Affiliations:** 1 Departamento de Fisiología, Biofísica y Neurociencias, Centro de Investigación y de Estudios Avanzados del Instituto Politécnico Nacional, Ciudad de México, 07360 México; 2 Unidad Periférica de Neurociencias, Facultad de Medicina, Instituto Nacional de Neurología y Neurocirugía “MVS”, Universidad Nacional Autónoma de México, Ciudad de México, México

**Keywords:** Neurological disorders, Neuroscience

## Abstract

Parkinson`s disease (PD) is the second most prevalent neurodegenerative disease, and different gene therapy strategies have been used as experimental treatments. As a proof-of-concept for the treatment of PD, we used SAM, a CRISPR gene activation system, to activate the endogenous tyrosine hydroxylase gene (*th*) of astrocytes to produce dopamine (DA) in the *striatum* of 6-OHDA-lesioned rats. Potential sgRNAs within the rat *th* promoter region were tested, and the expression of the Th protein was determined in the C6 glial cell line. Employing pseudo-lentivirus, the SAM complex and the selected sgRNA were transferred into cultures of rat astrocytes, and gene expression and Th protein synthesis were ascertained; furthermore, DA release into the culture medium was determined by HPLC. The DA-producing astrocytes were implanted into the *striatum* of 6-OHDA hemiparkinsonian rats. We observed motor behavior improvement in the lesioned rats that received DA-astrocytes compared to lesioned rats receiving astrocytes that did not produce DA. Our data indicate that the SAM-induced expression of the astrocyte´s endogenous *th* gene can generate DA-producing astrocytes that effectively reduce the motor asymmetry induced by the lesion.

## Introduction

Parkinson’s disease (PD) is the second most prevalent neurodegenerative disease, and its incidence increases with aging [1]. In idiopathic PD, the vulnerability and dysfunction of the dopaminergic neurons that causes their death produce an imbalance between the direct and the indirect basal ganglia pathways [2–4], causing motor symptomatology such as tremors, bradykinesia, rigidity, and postural instability that are characteristics of PD, and they occur when more than 75% of the dopaminergic neurons die [1, 5]. The pathophysiology of PD has been described as the death of the dopaminergic neurons of the *substantia nigra pars compacta* (SNpc); which produce the dopamine (DA) necessary for the correct motor control exerted by the basal ganglia (BG) [5]. Because of this, the gold standard for treating PD is the synthesis replacement of DA by the administration of its precursor, Levodopa (L-DOPA) [6, 7]. However, this treatment has a limited useful time of around five years. Furthermore, the use of L-DOPA does not stop the progressive death of the dopaminergic neurons and induces several problems such as dyskinesias (abnormal involuntary motor movements). The disease is associated with deficits in neurotransmitters other than DA that can result in non-motor symptoms, such as cognitive, psychiatric and sensorial symptoms (dementia, depression, anosmia, etc.) and gastrointestinal complications (constipation, drooling, dysphagia, and nausea) that occur as the disease progresses [8–10]. Current pharmacological, surgical and alternative PD treatments preserve the use of L-DOPA [5, 11].

Diverse promising gene and cell therapy strategies have been tested for PD. These include transplants of neurons derived from induced pluripotent stem cells (iPSc cells) [12]. Also, the expression of disease-modifying transgenes like neurotrophic factors (glial cell-line derived neurotrophic factor (GDNF), neurturin (NRTN), artemin (ARTN), and persephin (PSPN) or of non-disease modifying transgenes, including tyrosine hydroxylase (Th), and glutamic acid decarboxylase (GAD) have been tried with interesting results in experimental models, and some in early clinical protocols [13–16]. However, using viral vectors in some of these protocols may cause the integration of transgenes into the host genome, which may induce mutagenesis or activate the host immune response limiting the effectivity [17, 18].

Astrocytes are essential for the immunological response of the brain in PD, and their participation in that response has been observed in both the *substantia nigra* and the *striatum* in animal models and postmortem studies in PD patients [19]. There is an important reactive astrogliosis in PD, which indicates the relevant role of astrocytes in the response to the disease [20]. Astrocytes are resistant to insults and participate in the dopamine (DA) metabolism because they express both the amino acid transporter (LAT) and the DA transporter (DAT), allowing the uptake of L-DOPA and DA observed in the *striatum* [20, 21]. Moreover, astrocytes express aromatic L-amino acid decarboxylase (AADC), an enzyme that converts L-DOPA into DA. In cell culture studies, the capacity of astrocytes to convert L-DOPA to DA in a concentration-dependent manner has been demonstrated [22]. Thus, Th is the only enzyme that astrocytes require to produce DA. We previously showed that either implanted astrocytes or endogenous astrocytes expressing transgenic Th produce DA in the *striatum* causing behavioral recovery in 6-OHDA-lesioned rats and in a non-human primate MPTP model [16, 23, 24].

Clustered Regularly Interspaced Short Palindromic Repeats associated with Cas (CRISPR-Cas) has already changed the gene therapy field since it represents a highly efficient gene editing tool [25, 26]. Synergistic Activation Mediator (SAM) is a second-generation CRISPR system capable of activating gene expression using an enzymatically inactive Cas9 (dCas9) and co-transcriptional activators. This CRISPR-based system has demonstrated be a robust and efficient method to induce gene activation [27–29].

Therefore, in the present work, we performed a proof-of-concept experiment to test the use of SAM in gene therapy for the treatment of PD. This work aims to evaluate the effectiveness of the endogenous activation of genes to induce the synthesis of DA in cells of glial origin and the effects of its implantation in a murine model of PD. We propose that the endogenous expression of *th* from rat astrocytes induced by SAM will allow these cells to produce DA and cause behavioral improvement in the 6-OHDA hemiparkinsonian rat model. The therapy proposed here will avoid fluctuations due to dosing, absorption and penetration into the brain, and simultaneously spare multiple organ systems from exposure to L-DOPA and its metabolites. In the brain, it will spare large areas from the resulting conversion to DA, and spare specific neurons, such as serotoninergic neurons from the competition with tryptophan, both for transport from plasma and for decarboxylation. The alternative offered here presents a novel method to achieve this targeted therapy that will increment the drug-free period of treatment and can combine with other therapies in more advanced cases.

## Materials and methods

### Sequence and cloning

The lentiSAMv2 and lentiMPH v2 plasmids (a gift from Feng Zhang Addgene #75112 and #89308) were used for CRISPR-Cas9 activation. We analyzed the promoter region of the rat *th* gene, 1000–1200 bp upstream of the transcription start site, searching for PAM regions. We obtained ~20 nucleotides sequences that were evaluated using BLAST analysis to check for mismatched sites on the rat (*Rattus Norvegicus*) genome. In addition, the *th* promoter region was also assessed with E-CRISP, CRISPRESSO2, CHOPCHOP, and Benchling software to select the candidate sequences by specificity score, annotation score, efficiency score, mismatched score, and the number of hits. Finally, we chose 13 sgRNA candidates that were evaluated for mismatches using the BLAST tool (https://blast.ncbi.nlm.nih.gov/Blast.cgi). The single-guide RNA (sgRNA) sequences used in this study are listed in Supplementary Table 1, and were cloned in the lentiSAM v2 vector, employing the Golden Gate protocol using *BsmB*I (Thermo Fisher Scientific cat. no. IVGN0136) for plasmid restriction, t4 PNK (NEB, cat.no. M0201) for oligo annealing and t7 ligase (NEB, cat.no. M0318) for cloning [27]. The cloning sgRNA region was amplified by PCR using specific primers. The amplified PCR products were purified using the Sap-Exo kit. (Jena Bioscience, Cat. No. PP-218L, Germany) following the manufacturer´s instructions. The purified products were sequenced (forward and reverse) by automatic sequencing on an ABI PRISM 3100 with the Kit of BigDye Terminator v3.1 cycle sequencing (Applied Cat No. 4337458, Biosystems, USA). We show in Supplementary Fig. 1B the sequence of the chosen *th* sgRNA.

### Cell culture

HEK293T/17 cells (ATCC CRL-11268) were maintained in DMEM High Glucose (Gibco, cat. no. 12800-017) and pyruvate supplemented with 10% fetal bovine serum (FBS) (Gibco, cat. no. 16000-044) and 1% penicillin/streptomycin (Thermo Fisher Scientific cat. no. 15140122). Cells were incubated at 37 °C, 5% CO_2_ and 95% humidity. Cells were passaged every other day at a ratio of 1:6 or 1:5 using Trypsin 1x (Sigma, cat. no. 59428C). The C6 rat glioblastoma cell line (ATCC CCL-107) was maintained in F12 DMEM (Gibco, cat. no. 12500-062) with L-glutamine and pyruvate supplemented with 10% fetal bovine serum (Gibco, cat. no. 16000-044) and 1% penicillin/streptomycin (Thermo Fisher Scientific, cat. no. 15140122), incubated at 37 °C, 5% of CO_2_, and 95% humidity, passaged every other day at a ratio of 1:6 or 1:5 using Trypsin 1x (Sigma, cat. no. 59428C). CTX-TNA2 cells, a primary astrocyte non-tumorigenic line obtained from rat cerebral cortex (ATCC CRL-2006), was maintained in high-glucose DMEM (Gibco, cat. no. 12800-017) with pyruvate supplemented with 10% fetal bovine serum (Gibco, cat. no. 16000-044) and 1% penicillin/streptomycin (Thermo Fisher Scientific, cat. no. 15140122). Cells were incubated at 37 °C, 5% of CO_2_ and 95% humidity_,_ passaged every other day at a ratio of 1:6 or 1:5 using Trypsin 1x (Sigma, cat. no. 59428C).

### Transfection of C6 cells

C6 cells were transfected with Lipofectamine 3000 (Thermo Fisher Scientific, cat.no. L3000-15). The SAM system with each sgRNA was tested in C6 cells seeded in 60 mm Petri dishes; for each sequence cells were seeded at ~40% confluence (~1.2 ×10^6^ cells) the day before and 0.5 µgrams of plasmid DNA was transfected overnight with 1.5 ml of medium without serum and medium changed the following day with fresh medium with 10% FBS.

### Lentivirus production and cell infection

One day before transfection, HEK293T cells were seeded at ~50% confluency (~4.4 ×10^6^ cells) in 100 mm Petri dishes. Cells were transfected the next day at ~70–80% confluence (~6.6 ×10^6^ cells). For each petri dish, 3 μg of each of the following plasmids were transfected using 24 µL of Lipofectamine 3000 (Thermo Fisher Scientific, cat.no. L3000-15) and 24 μL of P3000 Enhancer (Therm Fisher Scientific, cat.no. L3000-15): pMD2.G (Addgene 12259); pMDLg/pRRE (Addgene 12251) and pRSV-Rev (Addgene 12253). Cells were transfected with 8 mL of high-glucose DMEM (Gibco 12800-017) without fetal bovine serum, and 12 h after the transfection, the medium was changed for complete maintenance medium. The virus supernatant was harvested 48 h post-transfection and centrifuged at 1200 rpm to eliminate debris and dead cells. The virus supernatant was then aliquoted and stored at −80  °C. Different cell lines were infected with a 0.3 viral titer on 1.5 ml of pseudolentivirus with the SAM system, and each sgRNA and incubated overnight; then, the medium was removed, and fresh medium was applied; after 48 h, cells were processed for RNA and protein extraction. Resistance curves for blasticidin S HCl (10 μg/ml, Thermo Fisher Scientific, cat.no. A1113903) and hygromycin B (300 μg/ml, Sigma cat.no. H3274) were constructed, and cells were selected by their resistance to antibiotics. After 24 h of infection, fresh media containing the antibiotics were applied, and cells were maintained under antibiotic selection.

### RT-PCR

Cells were seeded in 60 mm plates and grown to 90% confluency (~2.8 ×10^6^ cells) before RT-PCR. Total RNA was extracted using the Trizol reagent (Invitrogen, cat. no. 15596026). RNA quantification and purity were assessed by spectrophotometric analysis using the Nanodrop instrument (Thermo Fisher Scientific, cat. no. 13-400-525). DNAse I (Sigma, cat.no. AMPD1) was used to degrade DNA; cDNA was synthesized using M-MLV (Invitrogen, cat. no. 28025-013), dNTPs 100 mM (Invitrogen, cat. no. 10297-018), and oligo dT (T4 Oligo Oligo dT 18-mer).

RT-PCR was performed using Taq Polymerase Kappa (Sigma, cat.no. BK1004), dNTPs (Invitrogen, cat. no. 10297-018), UltraPure™ DNase/RNase Free Distilled Water (Invitrogen, cat. no. 10977015) and the following primers were used: for *th* (FW ‘GGAGAGCTCCTGCACTCC’ REV ‘GGCATAGTTCCTGAGCTTG’), for *β-actin* (FW ‘TCACGCACGATTTCCCTCTCAG’ REV ‘TGGCACCACACCTTCTACA’), for *dCas9* (FW ‘GCACATACCACGATCTGCTG’ REV ‘CGCTTCAGCTGCTTCATCAC’) and for *ms2* (FW ‘GGGATGTGACAGTGGCTCC’ REV ‘GGACCTCCACCTTGATGGTATAC’). (Sigma-Aldrich). We used the following temperatures for the PCR assays: 94° for denaturation, 57 °C for annealing, and 72° for the extension step using a T100 Bio-Rad thermal cycler.

### Western blot

Protein extraction from cells or tissue was performed using a lysis buffer that contained a protease inhibitor cocktail (Complete, Roche Diagnostics, cat. no. 04574834001). Total protein (25 μg) was loaded for each sample for separation by SDS–polyacrylamide gel electrophoresis (8%) and transferred onto nitrocellulose membranes (BioRad; cat. no. 1620115). Membranes were blocked with 5% non-fat milk diluted in Tris-buffered saline containing 0.1% Tween-20 (TBST) for one h and incubated in the presence of anti-Th (Abcam ab112 monoclonal anti-rabbit 1:1000), or anti-Th F-11 (sc-25269 monoclonal anti-mouse, 1:1000), or anti-Th Millipore ab152 (monoclonal anti-rabbit 1:1000), anti-Glial Fibrillary Acidic Protein (DAKO Z0334 polyclonal anti-rabbit 1:2000), anti-Beta-actin-peroxidase (Sigma A3854 monoclonal anti-mouse 1:25000). Proteins were revealed using secondary peroxidase-coupled anti-rabbit and anti-mouse antibodies (Jackson ImmunoResearch), using the Western Lightning Plus-ECL Kit (PerkinElmer; Waltham, MA). Images were digitally captured using the FUSION SOLO S instrument (Vilbert Smart imagining). Autoradiograms were analyzed by densitometry using the Image J^®^ software (NIH).

### Immunofluorescence

C6 and astrocytes were seeded on coverslips (~80% of confluence in a 60 mm petri dish, ~2.5 ×10^6^ cells), fixed with 4% paraformaldehyde and/or cold methanol, washed twice with PBS, and incubated using 0.2% Triton X-100/PBS/1% BSA for 30 min at room temperature. Cells were incubated using a primary mouse monoclonal antibody against anti-Th (Abcam ab112 monoclonal anti-rabbit) in C6 cells and anti-Glial Fibrillary Acidic Protein (DAKO Z0334 polyclonal anti-rabbit) and anti-Th F-11 (sc-25269 monoclonal anti-mouse) in astrocytes for 12 h. Coverslips were washed with 0.2% Triton X-100/PBS and incubated using the appropriate secondary antibodies, Alexa Fluor 594 donkey anti-mouse IgG (H + L) (Invitrogen A21203), and Alexa Fluor 488 donkey anti-rabbit IgG (H + L) (Invitrogen A21206.) Nuclei were counterstained using DAPI with Vectashield (Vector Laboratories).

### Experimental subjects

Male Wistar rats weighing ~200 g at the start of the experiment were used. Rats were housed on a 12-h light/dark cycle with free access to food and water at ~25 °C, with a relative humidity of 40–60%. All animal studies were performed according to the Guide for the Care and Use of Laboratory Animals [30], as adopted by the US National Institutes of Health and the Mexican Regulation of Animal Care and Maintenance (NOM-062-ZOO-1999). Rats were maintained and handled according to the guidelines of the CINVESTAV Animal Care Committee, making all efforts to minimize suffering and the number of animals used. The principles of the 3Rs (Replacement, Reduction, and Refinement) were followed to guarantee a humane endpoint.

### Brain tissue immunofluorescence

Rats were euthanized ten weeks after the transplant of astrocytes or the sham treatment with an overdose of sodium pentobarbital (200 mg/kg i.p.) and transcardially perfused with saline followed by 4% paraformaldehyde (PFA) in phosphate-buffered saline (PBS). Brains were dissected and post-fixed in PFA at 4 °C for 24 h and then cryoprotected in 10% sucrose/PBS for 24 h, 20% sucrose/PBS for 24 h, and 30% sucrose/PBS for an additional 24 h at 4 °C. Frozen coronal 35-μm-thick sections were cut in a sliding microtome (Leica Microsystems), collected in 2% PFA/PBS, and stored at 4 °C until processing.

Brain sections chosen for the analysis of the *striatum* were incubated in free-floating conditions in 0.2% Triton X-100/PBS for 30 min and blocked for 30 min in 1% BSA/0.2% Triton X-100/PBS at room temperature. Then, sections were incubated overnight with the primary rabbit antibody against the glial fibrillary acidic protein (Dako Z0334 polyclonal antirabbit GFAP, 1:500) and anti-Th (Sigma T1299 monoclonal anti-mouse, 1:200) diluted in 0.02% Triton X-100/0.1% BSA/PBS. For primary antibody detection, sections were rinsed with 0.02% Triton X-100/PBS and incubated for 1 h at room temperature with the secondary antibody Alexa Fluor 594 donkey anti-mouse IgG (H + L) A21203 and Alexa Fluor 488 donkey anti-rabbit IgG (H + L) A21206 diluted in the same solution as the primary antibody. Nuclei were counterstained using 4′,6-diamino-2-phenyldole (DAPI) with Vectashield (Vector Laboratories).

### Brain tissue immunohistochemistry

Immunohistochemistry was performed using a rabbit monoclonal anti-tyrosine hydroxylase Th antibody (1:400, Abcam ab112), a rabbit antibody against the glial fibrillary acidic protein (Dako Z0334 polyclonal antirabbit GFAP, 1:500) and a biotinylated anti-rabbit IgG (H + L) (1:100; Vector Laboratories, Burlingame, CA, USA). The immunohistochemical staining was developed using the avidin–biotin–peroxidase complex (1:10; ABC Kit; Vector Laboratories) and DAB (Sigma). Immunohistochemical labeling was observed with an Olympus BX53 microscope and images were digitized.

### Confocal microscopy

Triple-labeled images were obtained using a confocal laser-scanning microscope (Leica TCS-SP8) in the XYZ (Z-stacks) mode using a 63X (oil immersion) objective. The following excitation lasers/emission filters settings were used for the various chromophores: an argon laser was used for the Alexa Fluor 488, with a peak excitation at 490 nm and emission in the 505–530 nm range; a He-Ne laser was used for the Alexa Fluor 594 with a peak excitation at 543 nm and emission in the 568–615 nm range; and a UV laser was used to reveal DAPI with a peak excitation at 456 nm and emission in the 410–480 nm range, using the sequential acquisition of separate wavelength channels to reduce interference between channels. The Z-stacks (3–4 optical slices) were then converted into a three-dimensional projection image using the Leica LAS AF lite software.

### High-performance liquid chromatography (HPLC)

Brain slices, 100 µm thick, were cut using a Leica microtome (RM2125 RTS) in ice-cold Krebs-Henseleit solution. Immediately, left and right *striata* sections were dissected and collected in an amber 1.5 ml Eppendorf tube with sterile PBS solution at 4 °C (100 µl) and 50 µl of perchloric acid 0.1 N, the tissue was manually homogenized, sonicated and ultracentrifuged, the supernatant was filtered through a 0.22 µm filter (Millipore GSWP04700) to assay for DA and DOPAC [31, 32]. The medium culture samples were collected in an amber 1.5 ml Eppendorf tube with 50 µl of perchloric acid 0.1 N per 950 µl of medium, samples were shaken with a vortex for a minute and ultracentrifuged, the supernatant was filtered through a 0.22 µm filter (Millipore GSWP04700) to assay for DA and DOPAC. Briefly, dopamine levels were determined using an HPLC method with electrochemical detection as previously described [33, 34]. The separation of Dopamine and DOPAC was performed using a dC-18 microbore column (Atlantis, 2.1 ×150 mm, Waters Co.). The mobile phase was: buffer 97%, NaCl 135 mg/l; citric acid 10.5 mg/l; EDTA 20 mg/l; OSA 20 mg/l; methanol 3%, pH 2.9 adjusted with NaOH. The flow rate was 0.3 ml/min at 30 °C. The electrochemical detection system was an Intro; Waters Co. coupled to a glassy carbon electrode (VT-03, Antec Leyden). The oxidation potential was þ380 mV vs. silver/silver chloride reference electrode (ISSAC).

### 6-OHDA stereotaxic lesion surgery

Rats were anesthetized with Ketamine 75 mg/kg and Xylazine 5 mg/kg i.p; placed on a David Kopf stereotaxic frame and injected unilaterally with 6-hydroxydopamine (6-OHDA by Sigma, cat. no. H4381); 16 μg/μl of saline containing 0.1% ascorbic acid) in the medial forebrain bundle at coordinates (AP −1.8; ML 2.4; DV −7 mm) relative to Bregma according to the rat brain atlas of Paxinos and Watson [35], and 5 µl were administered at a rate of 1 µl per minute. To prevent noradrenergic neuron damage, rats were pre-treated with desipramine (10 mg/kg i.p.) 40 min before the surgery. Twelve days after the 6-OHDA lesion, only rats showing ten or more ipsilateral turns/min 30 min after amphetamine injection were included in the study [36]. A group of sham-lesioned rats with the surgical procedure but no stereotaxic lesion or administration of 6-OHDA was used as a control. We call lesioned side the right side receiving 6-OHDA and as the intact side the left side that did not receive stereotaxic lesion and 6-OHDA administration.

### Implantation of astrocytes

Hemi-parkinsonian rats (~300 g) were anesthetized with Ketamine/Xylazine (75/5 mg/kg i.p.) and placed on a David Kopf stereotaxic frame and received 20,000 astrocytes in each of the two sites in the lesioned *striatum*: anterior (AP 1.9; ML 2.2; DV −5) and posterior (AP 0.9; ML 3.0; DV −4) relative to Bregma according to the brain atlas of Paxinos and Watson [35]. Astrocytes (AST) and astrocytes expressing Th (AST-TH) were trypsinized from 90% confluent 60 mm Petri dishes, counted, and 20,000 cells were immediately concentrated in 3–4 µl of culture medium without serum to be used for the implant procedure [37]. Sham-lesioned rats did not receive implants.

### Behavioral tests

Rats were trained for two weeks before the 6-OHDA lesion. Two weeks after the 6-OHDA lesion, astrocytes and AST-TH were implanted in the *striata* of experimental subjects. Implanted rats were evaluated for the following nine weeks. Experimental and control rats were sacrificed ten weeks after the lesion to obtain *striatal* tissue for HPLC, immunofluorescence, and western blot assays.

### Amphetamine-induced rotations test

Rats were challenged with amphetamine (8 mg/kg i.p. Sigma, cat. no. NMID420D) and tested for ipsilateral circling behavior; the number of turns per minute was measured and recorded for 30 min, 15 min after the peritoneal administration of amphetamine [38].

### Cylinder test

The test was performed as follows: Rats were placed individually inside an acrylic cylinder (diameter, 22 cm; height, 26 cm) with a mirror behind it at a 45° angle to allow 360° vision. Rats were video recorded for 5 min after they first touched the cylinder walls with either the impaired or unimpaired forelimb or both simultaneously. Scores were calculated by the asymmetry ratio: right-left/ (right + left + both). Scores on the forelimb asymmetry ratio range from −1 to 1 [39, 40].

### Inclined beam balance test

Animals were trained for three days to walk along 2 m long beams from the starting platform to a cage on the upper part; animals were presented with a negative stimulus in the base of the beam as a yellow light lamp and a sugar cookie reward in the dark cage. The beams were inclined at 15°; two different beams with 24- and 18-mm widths were used. The animals were tested for the basal measure four days after the start of the training before the 6-OHDA lesion. The test recorded the time it took the animals to walk from the base of the beam to the cage at the end of the shaft. Only animals that completed the test in less than 120 s during the basal determination were included in the experiment [41].

### Distribution of implanted astrocytes

Three 6-OHDA-lesioned Wistar rats were transplanted with AST-TH and ten weeks after implantation were euthanized, and *striatal* coronal sections obtained (~30 µm) from AP +1.60 mm to −0.92 mm. Sections were stained for GFAP, Th and counterstained with DAPI to reveal the nuclei. The estimated coordinates were established using the Paxinos and Watson rat brain atlas [42]. We determined the number of GFAP^+^/TH^+^ cells of three independent fields of each *striatal* slice from three rats.

### Statistical analysis

Results are expressed as mean ± SD (*n* = 4). The sample size was calculated with G*Power software (Faul, Erdfelder, Lang and Buchner, 2007); a large effect size was used. We used the GraphPad Software version 8.0 to analyze the data. We evaluated data distribution with the Shapiro–Wilk test. Dopamine HPLC determination was analyzed with Brown–Forsythe ANOVA and Welch´s ANOVA test with a two-stage linear step-up procedure of Benjamini, Krieger, and Yekutieli test as *post hoc*. DOPAC/DA ratio and Th densitometry comparison were analyzed by One-Way ANOVA and Tukey´s multiple comparations test as post hoc. *P* < 0.05 was considered statistically significant.

For the behavioral tests we used four animals *per* group, after applied inclusion criteria based on the results of the test training, animals were randomized place in each group. The study was blinded during the data analysis. Amphetamine-induced rotation test data were analyzed with a two-way ANOVA with Sidak´s multiple comparisons *post hoc* test. Forelimb placement asymmetry, and the inclined beam motor balance test (24 and 18 mm) data were analyzed with Repeated Measures ANOVA and Tukey´s multiple comparison *post-hoc* test. *P* < 0.05 was considered statistically significant, variance between the compared groups was estimated, and it was similar.

## Results

### CRISPR activation screening for Th expression

**Fig. 1:**
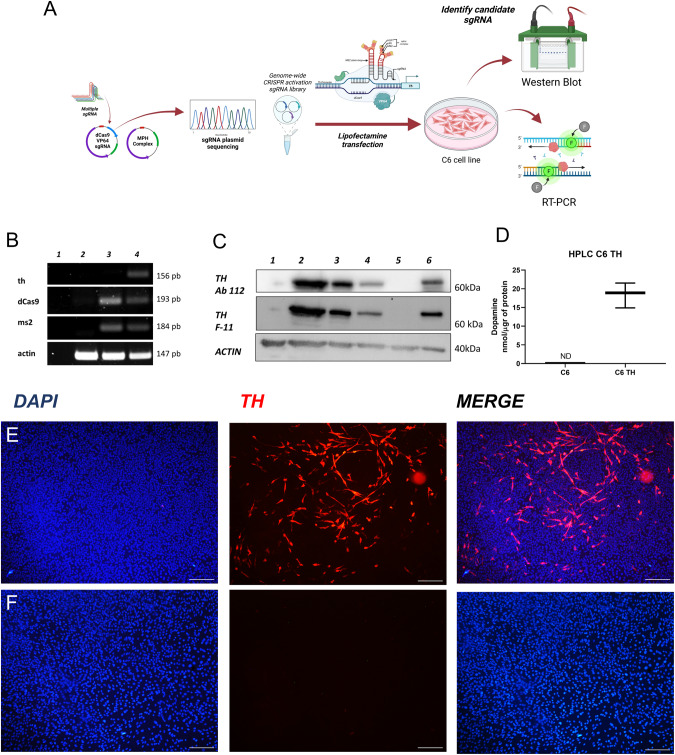
Tyrosine hydroxylase (Th) expression in C6 cells induced by the SAM CRISPR system. **A** Diagram of experimental design for the SAM system. **B** Detection of *th* mRNA in C6 TH4 cells, as determined by RT-PCR; lane 1 H2O control, lane 2- C6 cells, lane 3– C6 cells transfected with SAM system and lane 4- C6 cells transfected with the TH4 sgRNA. **C** Detection of the Th protein, as determined by Western blot analysis; lane 1- C6 cells, lane 2 to 4- C6 TH4 (different samples), lane 5- C6 SAM and lane 6- Rat *striatum* sample. **D** Determination of DA in the culture media, as determined by HPLC C6 cells and C6 TH4 cells; independent t-test *p* = 0.0007, *n* = 3. **E** Immunofluorescence for Th in C6 TH4 cells. **F** Immunofluorescence for Th in C6 cells. Bars represent 20 µm, nuclei revealed with DAPI.

We analyzed the promoter region of the rat genome using the DNA sequence from the Gene library of NIH (Gene ID: 25085, *Norway rat* chromosome 1) to determine potential *th* sgRNAs, as described in the methodology. We chose sequences with the highest specificity score, annotation score, efficiency score, and a high number of hits but non-genome mismatches (other genome sites where the oligo can align differently to the specific region). These sgRNAs were analyzed with the BLAST tool to check for mismatches in other rat genome regions (*Rattus Norvegicus*) and sgRNAs that did not align with other areas of the rat genome were selected (Fig. 1A). Thirteen sgRNAs were chosen to activate the rat *th* gene (Supplementary Table 1) and were individually cloned in The SAM v2 vector (Addgene #75112) using The Golden Gate protocol [27]. To test the selected sequences, C6 cells were transfected with the SAM system with each *Th* sgRNA, protein expression induced by each sgRNA transfected, are shown in Supplementary Fig. 1A.

The 13 th sgRNA sequences were tested, but we only show the results from TH4 sgRNA since this sequence achieved the highest levels of Th protein expression. The selected *th* sgRNA was sequenced to verify its sequence (Supplementary Fig. 1B).

RT-PCR was used to evaluate the levels of the *th* mRNA in C6 cells transfected with the selected *th* sgRNAs and, as a positive control, the rat *striatum* was employed. We did not find *th* mRNA expression in C6 control cells or those transfected with SAM system without *th* sgRNA (Fig. 1B, lane 3), *th* expression was only observed in C6 cells transfected with the complete SAM and the *th* sgRNA (Fig. 1B, lane 4). Moreover, we also determined the expression of the Th protein in the C6 transfected cells using two monoclonal antibodies (Abcam 112 and Santa Cruz Technologies F-11). In Fig. 1C, we show the blot of C6 cells transfected with the SAM system together with TH4 sgRNA, and it can be observed that only these cells express Th (Fig. 1C, lanes 2 to 4), in contrast we found no expression of Th in control (lane 1) or C6 cells transfected without *th* sgRNA (lane 5). As a positive control for the expression of Th, we used rat *striatum* (lane 6). In C6 transfected cells selected with blasticidin and hygromycin, DA production was detected. The stable C6 Th-expressing cell line released dopamine (DA) to the culture medium but DA could not be detected in the medium from control C6 cells (Fig. 1D). The double antibiotic selection was applied to ensure that all elements of the SAM system and the selected *th* sgRNA were stably expressed.

Then we showed the expression of Th by immunofluorescence in C6 cells with the SAM system transfected with or without the *th* sgRNA (Fig. 1E, F). As can be seen in cells that express *th* mRNA, a positive stain for Th was observed, in contrast to C6 cells that do not express the messenger. These data demonstrate that the SAM-TH system can activate the expression of the endogenous *th* gene in cells of glial origin and induce the synthesis of an active protein that can produce and release DA to the extracellular medium.

### Expression of Tyrosine hydroxylase in cultures of rat astrocytes

**Fig. 2:**
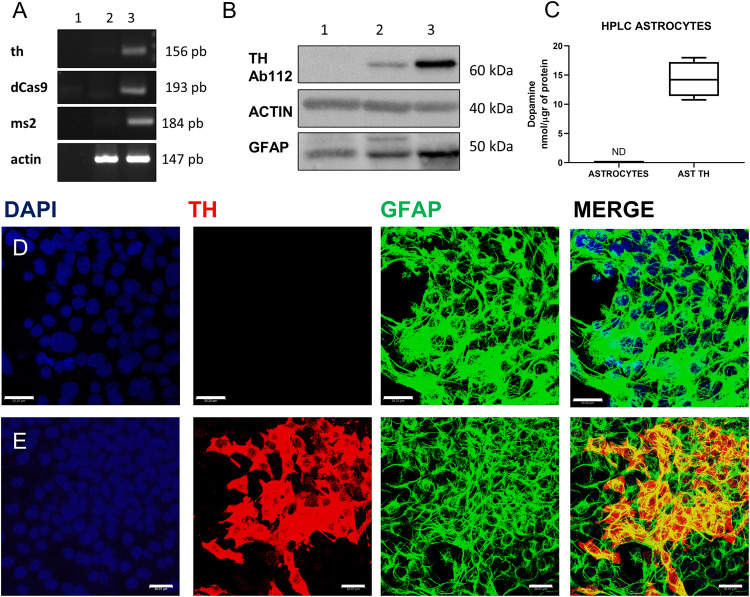
Expression of Th in astrocytes. **A** Detection of *th* mRNA and the components of the SAM system as determined by RT-PCR in astrocytes; lane 1- H2O (negative control), lane 2- astrocytes, lane 3- astrocyte-TH cells. **B** Detection of Th protein as determined by Western blot analysis; lane 1- astrocytes, lane 2- astrocyte TH, and lane 3- rat *striatum*. In **A** and **B**, actin is a positive control. **C** Determination of DA in the extracellular medium, as determined by HPLC in astrocytes and astrocyte-TH cells; independent t-test *p* = 0.01, *n* = 3. **D** Immunofluorescence in astrocytes for Th, GFAP, and DAPI (Nuclei). **E** Immunofluorescence for Th, GFAP, and DAPI in astrocyte-TH cells. Images in **D** and **E** were obtained with confocal microscopy. Bars represent 30 µm.

A cortical astrocyte cell line was infected with the p-Lenti SAM TH and the MPHv2 complex, cells selected with blasticidin and hygromycin for 14 days and RNA and proteins were extracted from the cells. Figure 2A shows the expression of the *th* gene and of dCas9, and ms2 mRNA in infected astrocytes as assessed by RT-PCR. We did not detect *th*, dCas9 or ms2 mRNAs in astrocytes cells (Fig. 2A, lane 2), but they were expressed in astrocytes infected with the complete system (Fig. 2A, lane 3); from now on we will refer this cell line as AST-TH. We also determined the expression of the Th protein in infected astrocytes using a monoclonal antibody against Th (Abcam 112). In Fig. 2B, we show the blot of the Th protein of AST-TH cells, and it can be observed that these cells express Th (Fig. 2B, lane 2). As expected, no signal for Th was detected in control astrocytes (lane 1). As a positive control, we used the same amount of protein from the rat *striatum* (25 µg, lane 3). Moreover, in cultures of infected astrocytes, we determined the production of DA, and observed that the stable AST-TH-expressing cell line released DA to the culture medium (Fig. 2C), yet no detectable DA was observed in the medium of the culture of cortical control astrocytes. We then showed the expression of Th by immunofluorescence in AST-TH cells and in astrocytes (Fig. 2D, E). It can be seen that AST-TH cells stain for Th; in contrast, control astrocytes do not express the protein. The viral titer used to infect astrocytes was 3.0 with an infection efficiency of 26.8 ± 3% (*n* = 3).

### Transplanted Th-producing astrocytes (AST-TH) induce motor recovery in unilaterally 6-OHDA-lesioned rats

**Fig. 3:**
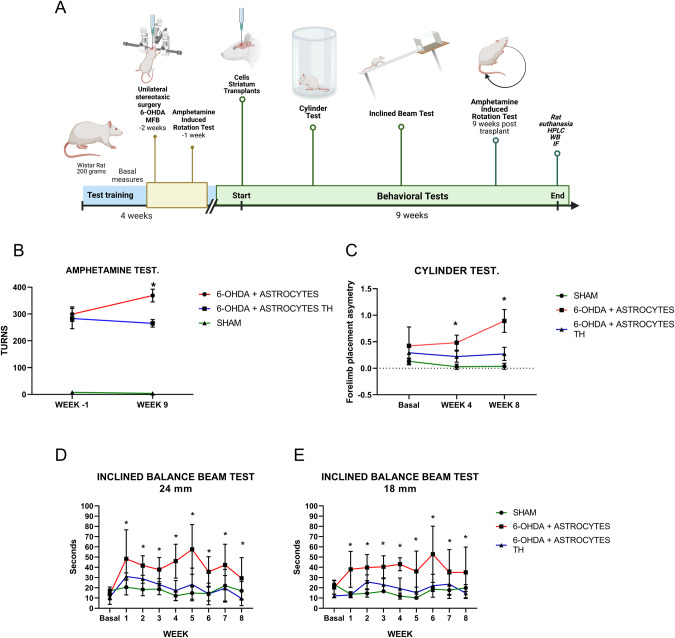
Behavioral tests of lesioned and implanted subjects. **A** Timeline of the experimental procedure. **B** Amphetamine-induced rotation test, one week before the implantation of astrocytes and nine weeks after the transplant; Sham, 6-OHDA + Astrocytes and 6-OHDA lesion + Astrocytes Th groups were compared with a Two-Way ANOVA test and Sidak´s multiple comparisons test was applied as *post hoc* test, *p* < 0.05 was considered statistically significant. **C** Forelimb placement asymmetry test, the basal level before 6-OHDA lesion, four and eight weeks after transplantation. Sham, 6-OHDA + Astrocytes and 6-OHDA lesion + Astrocytes Th groups were compared with a Repeated Measures ANOVA and for the three groups Tukey´s multiple comparison test as *post hoc* test was used as well as for each temporal determination, *p* < 0.05 was considered statistically significant. **D** Inclined beam motor balance test (24 mm width). **E** Inclined beam motor balance test (18 mm width). Sham, 6-OHDA + Astrocytes and 6-OHDA lesion + Astrocytes Th groups were compared with a Repeated Measures ANOVA and for the three groups Tukey´s multiple comparison test as *post hoc* test was used as well as for each temporal determination, *p* < 0.05 was considered statistically significant. Bars represent the Standard error of the mean (SEM), *p < 0.05, *n* = 4.

We tested whether AST-TH could induce motor control improvement in hemiparkinsonian rats. We used the amphetamine-induced circling, cylinder, and inclined balance beam tests to evaluate motor recovery. Figure 3A shows the timeline of the training and behavioral tests applied to the three experimental groups, which were followed for 13 weeks. A sham group was included in the experimental design to compare the magnitude of recovery. Lesioned rats were separated into two transplanted groups; one received AST-TH cells, and the other only primary astrocytes. Astrocytes were implanted in two different sites in the lesioned *striatum*. Nine weeks after cell implantation, the rotation test in response to amphetamine was repeated; results are shown in Fig. 3B. We found a significant increment in the circling behavior of rats receiving unmodified astrocytes compared with those receiving AST-TH cells. Sham-lesioned rats showed negligible turning behavior, and it did not change in the course of the experiment (Fig. 3B).

The cylinder test was applied to the three groups before the lesion to determine the basal asymmetry index. We observed that before the lesion or simulated surgery (sham), the rats did not show significant differences in forelimb placement asymmetry (FPA, Fig. 3C); in fact, values in the three groups were lower than 0.5, indicating low or no asymmetry in forelimb use. Then, we repeated the test 4 and 8 weeks after transplants, as previously reported [39, 40]. Four weeks after the transplants, rats treated with control astrocytes showed an increment of FPA over the values of the sham and AST-TH transplanted rats. This value increases significantly over 0.5 by the eighth week, whereas it remains under 0.5 in the other two groups, indicating that the AST-TH cell transplant prevents increments in FPA (Fig. 3C).

The training for the inclined beam balance started ten days before the 6-OHDA lesion; after it, a basal line was established for the three groups. Figure 3D, E show the results of the tests performed every week after transplantation (week 1–8). In Fig. 3D, the AST-TH transplanted group shows similar time climbing the beam compared with the sham group. In contrast, the primary astrocytes transplanted group increased the time needed to climb. Then, we used an 18 mm thick beam allowed us to measure fine motor control and balance. We observed no differences in the time it took the rats to climb the beam for the sham and the AST-TH group. However, the rats that received only astrocytes increased time scores and were statistically different from the other two groups (Fig. 3E). These data indicate that the implant of AST-TH cells decreases the motor imbalance and asymmetry of 6-OHDA-lesioned rats.

### Dopamine metabolism determined in implanted rats

**Fig. 4:**
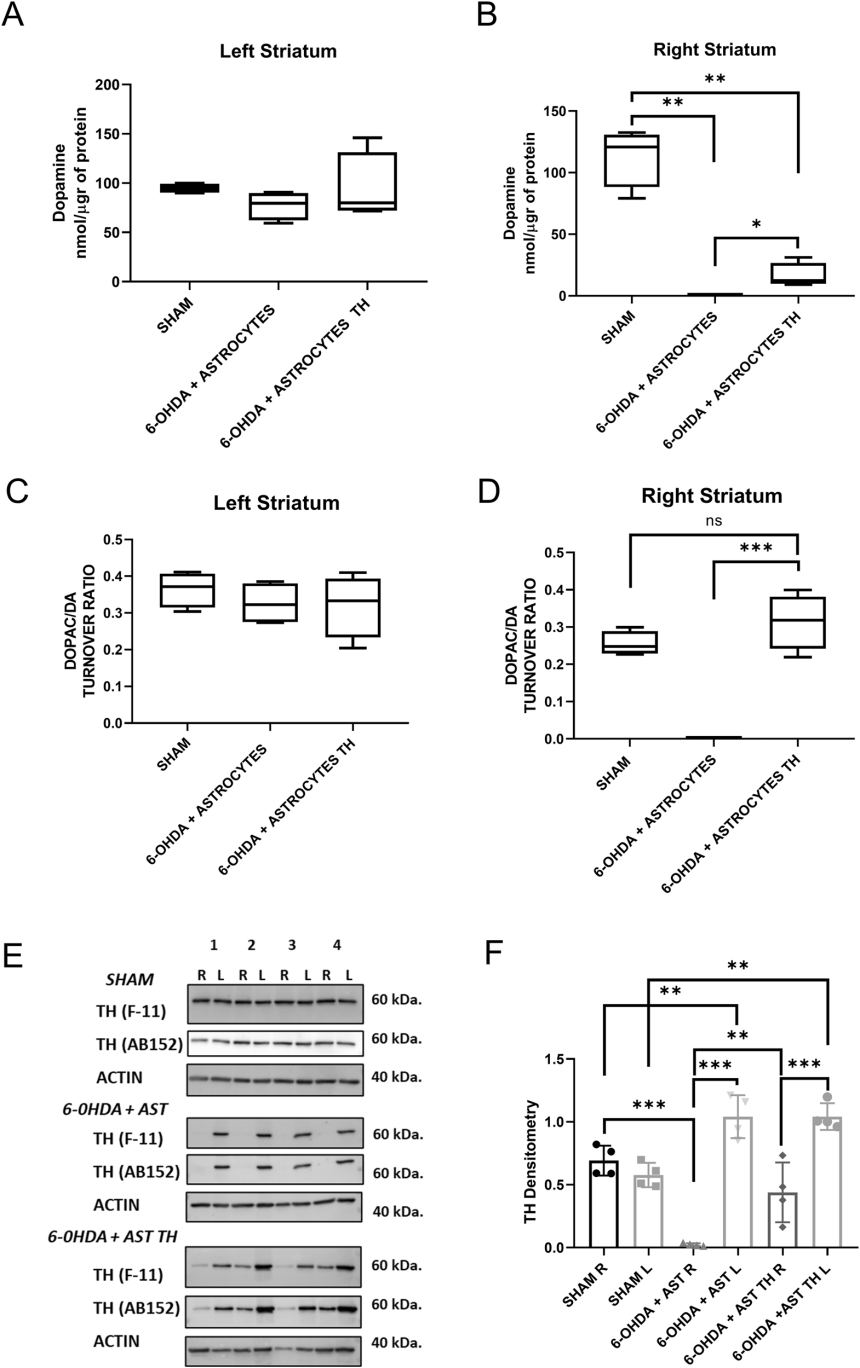
Levels of DA and DOPAC and expression of in the *striata* of rats. **A** Levels of DA, as determined by HPLC, normalized by µg of protein in the left (intact) and **B** in the right (lesioned) *striatum*. Brown–Forsythe ANOVA and Welch´s ANOVA test with a two-stage linear step-up procedure of Benjamini as post hoc. **C** DOPAC/DA ratio in the left (intact) and **D** in the right (lesioned) *striatum*. One-Way ANOVA and Tukey´s multiple comparisons test as post hoc. **E** Th protein, as determined by Western blot analysis using two different antibodies, and actin as a loading control. **F** Densitometric analysis of the expression of Th in *striata*. One-Way ANOVA and Tukey´s multiple comparisons test as post hoc. Bars represent Standard Deviation (SD) *p < 0.05, **p < 0.01, *** is *p* < 0.001; no significative difference (ns); *n* = 4.

We evaluated DA content and the DOPAC/DA turnover ratio in the different experimental groups, both in the lesioned and the intact *striatum*. Figure 4A shows that there were no DA content differences in the intact side of any of the groups; however, in the lesioned side (Fig. 4B), we found that DA cannot be detected in the lesioned-side of rats that received just astrocytes, whereas there is a clearly detectable level of DA in the *striatum* implanted with AST-TH. Then, we evaluated the turnover of DA in the *striatum*. In the left, control *striatum*, turnover values were similar in all groups. In contrast, no turnover can be determined in the control astrocytes transplanted group on the lesioned side. However, the turnover in the AST-TH group shows values that are not different from the sham group or intact *striatum*, suggesting an accelerated DA metabolism in the AST-TH implanted *striatum*.

Besides, we evaluated whether AST-TH cells still expressed Th protein in the transplanted *striatum*. Figure 4E shows the expression of Th as determined by western blot analysis from the homogenates used to determine DA content in the three groups of animals. It can be observed that homogenates from the *striata* of all the rats express the Th protein in the intact (left) side and that only in the sham and AST-TH groups, Th was detected in the lesioned (right) side. Densitometric analysis of Th expression using ab152 antibody indicates that in the intact side of control astrocytes and AST-TH groups, an increment of Th expression occurs (Fig. 4F); also, in the lesioned side of the AST-TH group, there is a recovery of Th expression comparable to the sham group. We can observe in the intact side of the transplanted groups a significant increase of Th expression in comparison with the Sham group; this effect has been attributed to a cross hemispheric nigrostriatal branching that occurs in the unilateral 6-OHDA lesion on the MFB, so dopaminergic fibers migrate to the contralateral *striatum* [43, 44].

### Expression of Th and GFAP in the lesioned striata

**Fig. 5:**
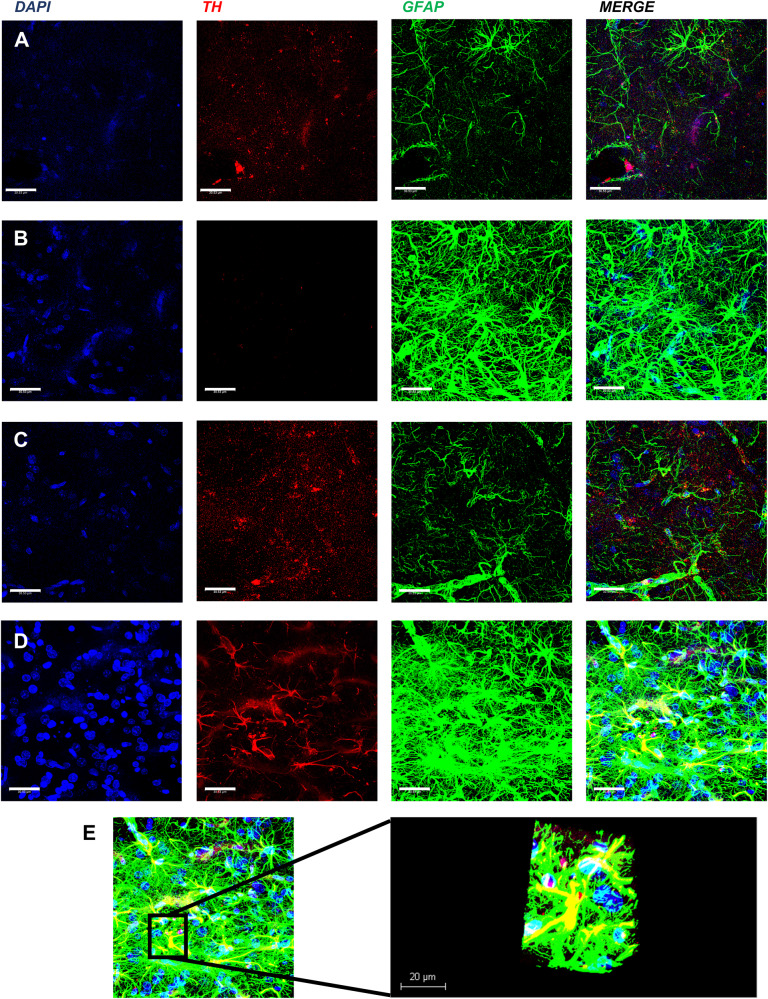
Expression of Th and GFAP in the *striata*, as shown by confocal immunofluorescence. Panels on row **A** show 6-OHDA + AST left side *striatum* (intact side), row **B** shows 6-OHDA + AST right side *striatum* (lesioned side), row **C** shows 6-OHDA + AST-TH left side *striatum* (intact side), and row **D** shows 6-OHDA + AST-TH right *striatum* (lesioned side). Th is revealed with a red fluorescent signal, and GFAP with a green fluorescent signal, DAPI (nuclei) in blue. Cells expressing both Th and GFAP show a yellow signal. **E** 3D reconstruction of Z-slices of an AST-TH implanted in the lesioned *striatum*. Bars represent 30 µm.

These series of experiments were designed to show the expression of both Th and GFAP in the implanted astrocytes. Figure 5 shows the expression of Th and GFAP in the intact (row A) and in the lesioned *striata* (row B) of rats receiving control astrocytes. Astrocytes can be observed in A and B, however, the TH signal can only be seen in the intact side, but not overlapping with the GFAP stain (see merge). Rows C and D show the same markers in rats implanted with AST-TH in the *striatum*. Astrocytes were observed on both sides. However, unlike the control astrocytes treated group (row D), Th stain in the lesioned side co-localizes with GFAP. In contrast, in the left control side, staining is like in the control astrocytes-treated group (compared to row A). Row E shows the Z-reconstruction of an astrocyte expressing both GFAP and Th from D row, indicating that the co-localization of Th and GFAP occurs in astrocytes. We also show DAB immunohistochemistry images of Th and GFAP to show the overall Th and GFAP staining on the *striatum* (Supplementary Figs. 2, 3). Supplementary Fig. 3A, B show a magnification of immunohistochemistry (IH) Th images of right and left *striata*, respectively.

### Distribution of AST-TH in the implanted *striatum*

**Fig. 6:**
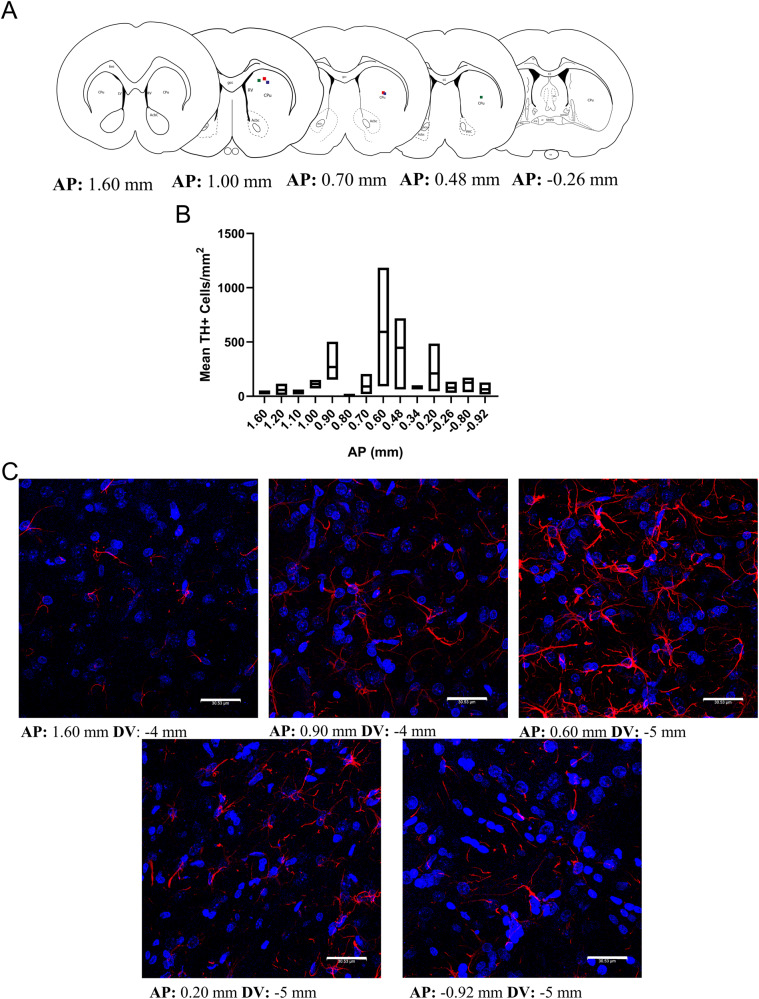
Distribution of AST-TH in implanted *striatum*. **A** Sequential coronal diagrams show the localization of the implants in the lesioned *striatum*. **B** Graph shows TH+ astrocytes/mm^2^ means ± SEM). **C** Confocal microscopy images showing implanted astrocytes at different levels of the *striatum*, as demonstrated by double labeling for Th (red) and DAPI (nuclei-blue). Bars represent 30 µm.

Finally, we analyzed the distribution of AST-TH cells within the *striatum*. To accomplish this goal, we obtained coronal serial sections of the *striatum* from AP + 1.60 mm to −0.92 mm, stained for Th, and counterstained with DAPI to reveal the nuclei. Figure 6A shows a series of progressive anteroposterior drawings of coronal sections of the left *striatum* level in which color squares indicate the approximate site of AST-TH cell injection. Mean of Th positive cells from the three subjects, along the stereotaxic coordinates, is shown in Fig. 6B. In Fig. 6C, it can be observed that all Th-positive cells in the lesioned and implanted *striatum* have an astrocyte-like morphology (Supplementary Fig. 5) and that they can be found widely distributed within the *striatum*, although with a higher concentration closer to sites of implantation (Fig. 6B, C). Moreover, to compare astrogliosis between the intact side and the lesioned side that received AST-TH, immunofluorescence against Th and GFAP was performed in different sections (Supplementary Fig. 6). And we can observe strong astrogliosis in the lesioned side (as well in Supplementary Fig. 4A, we can also observe an increase of the number of astrocytes on the lesioned *striatum* on DAB IH images at 4x, 10x and 20x), and AST-TH astrocytes expressing Th, but there are also GFAP positive cells, not expressing Th, indicating they are endogenous astrocytes. Finally, no Th positive astrocytes were observed on the intact side, although TH positive terminals are detected in the intact side.

## Discussion

This work is a proof-of-concept experiment using a CRISPR-Cas9 system to treat a neurodegenerative disease. The strategy of this experiment is based on the fact that the expression of Th induces the synthesis of DA in astrocytes since it is the only enzyme absent in astrocytes to complete the biosynthetic pathway to produce DA [23, 24]. We had previously demonstrated that the transfer of the *th* gene into astrocytes causes the expression of the enzyme, and motor recovery in both rats and in a non-human primate model of PD [16, 23, 24], thus demonstrating that astrocytes expressing Th can induce behavioral recovery in different animal models of PD. In the present work, we used a new and different approach: activating the endogenous astrocyte *th* gene. SAM induces higher transcriptional levels than other transgene transfer systems (retroviral and lentiviral methods), and even when compared to other CRISPR activation systems; it is highly specific; it does not disrupt the genome or cause double-strand breaks (DSBs), and the epigenomic modification is reversible [28, 29].

Moreover, third-generation pseudo-lentivirus (p-lenti) are an efficient and safe gene delivery system to obtain stable cell lines expressing SAM. Furthermore, the lentiviral system allows the delivery of multiple sgRNAs potentially obtaining the regulation of several genes within the same cell. Finally, these viruses are self-inactivating and less immunogenic than previous methods of viral gene transfer, and thus it is a safer method to be used in gene therapy protocols [45, 46].

Employing the SAM co-transcriptional complex, we activated the expression of the endogenous *th* gene in cells of glial origin, C6 glioma cells and rat astrocytes, which synthesized the protein, produced, and released DA into the medium. Furthermore, when the DA-producing SAM-engineered primary astrocytes were implanted in a rat PD model, they could reverse motor behaviors induced by the 6-OHDA lesion (Fig. 3). The motor improvement is attributable to the DA produced by astrocytes expressing their endogenous *th* gene, since no behavioral motor improvement was observed when astrocytes that did not activate the gene were implanted.

We also demonstrated the expression of the *th* gene in astrocytes by double labeling with the glial marker GFAP and Th. Furthermore, tyrosine hydroxylase protein was only detected in the lesioned *striatum* of rats implanted with Th-producing astrocytes, which also contained significant amounts of dopamine, that was absent in lesioned rats that received control astrocytes and did not express the *th* gene. All these data demonstrate that it is possible to induce the expression of the endogenous *th* gene of astrocytes with a CRISPR activation system and induce behavioral improvement in an PD animal model.

The levels of DA in the lesioned *striata* of rats receiving AST-TH were lower than those of control non-lesioned animals. There is a wide variation in the literature regarding the amount of DA necessary to induce behavioral recovery after *striatal* lesions, which can be 11% of those of normal rats [47, 48]. Thus, the levels shown in this paper (14.42%) are within the range previously reported to induce behavioral recovery. The effect induced by lower DA concentrations is probably due to the increased extracellular lifetime of DA likely caused by poorer DA reuptake caused by a reduction of DA transporters on the lesioned side, and by the hypersensitivity of DA receptors [49–51]. We consider that DA production could be increased by implanting more DA-producing astrocytes in the lesioned rats. We chose the first week after the 6-OHDA lesion to select the animals based on their turning behavior, since one week after the lesion the dopaminergic degeneration is sufficient to induce motor impairment, yet, the complete damage is observed between the third- and fourth-weeks post-lesion [52–54].

Several arguments support the use of astrocytes as a platform for producing dopamine and other molecules that may impact the development and treatment of PD, such as BDNF and GDNF, that can help preserve the few dopaminergic neurons remaining and improve the dopamine metabolism in the *striatum* [55, 56]. Previous studies demonstrated that astrogliosis is present in the site of the lesion in the 6-OHDA hemiparkinsonian model, and other animal models and postmortem PD studies have found inflammation on the affected *striatum*, specifically astrogliosis [20, 57]. In Fig. 5, on the lesioned sites of both groups, we can observe astrogliosis in the *striatum* but not in the intact side, this indicates that the implant of astrocytes in both the lesioned and transplanted groups have the same glial response. Astrogliosis and increases of GFAP expression have been reported in previous works after both and short and long periods after the lesion [58, 59].

Astrocytes can synthesize DA from L-DOPA and release it into the medium. Thus, the expression of Th in astrocytes is sufficient for the production and release DA from activated cells [22, 60]. Moreover, astrocytes possess DA reuptake mechanisms to regulate the levels of extracellular DA, express DAT (which recycles the dopamine back to the cell), and COMT/MAO (which metabolizes dopamine) [21]. For all these reasons, astrocytes are an excellent option to express Th and regulate DA levels in the basal ganglia. We chose the CTX-TNA2 rat astrocyte line because the double antibiotic selection necessary to create a stable cell line that expresses the SAM complex requires several cell passages. This astrocyte line is non-tumorigenic, and thus it is not rejected by the host. We observed (Fig. 6) the distribution of cells in the lesioned *striatum* nine weeks after implantation. Cells appeared healthy and with an extensive distribution in the tissue, which seems to follow a gradient from the injection sites.

The 6-OHDA hemiparkinsonian model is a late-stage PD, since it causes the unilateral death of the dopaminergic neurons, thus one hemisphere is depleted of DA. The lesioned rats present imbalance and motor asymmetry, as the lack of DA induces a loss of motor control from the lesioned side [61]. In our study, we wanted to determine whether DA released from AST-TH cells could correct the imbalance and asymmetry induced by the 6-OHDA lesion. To determine the motor alterations induced by the implant, we used a pharmacological test, the amphetamine-induced rotation sensitive to the release of dopamine from the nigrostriatal terminals and the sensitivity of the postsynaptic DA receptors. The amphetamine test allows us to evaluate the motor asymmetry improvement induced by the transplant, as previously shown [62–64].

To evaluate other aspects of motor behavior that the transplants can improve, we decided to perform a spontaneous test (cylinder test) and a test that requires training (inclined beam test). The cylinder test evaluates the asymmetry and the sensory-motor function that present the lesioned rats, and the beam test is a balance, sensory-motor, and motor performance evaluation. Rats that received AST-TH showed improvement in all tests, thus showing that the SAM-modified astrocytes, capable of expressing their endogenous *th* gene, are functional in an in vivo model of PD.

We chose the SAM system as a first approach of a proof-of-concept experiment because it is robust, stable and has a high capacity to activate endogenous genes in mammalian cells [65], multiple gene transcriptional activation [27], and the generation of iPSC from human fibroblast [66]. Moreover, cells modified using SAM have been used in a mouse model of metabolic syndrome with good results [67]. This indicates that SAM can be used to reprogram other mammalian cells, besides astrocytes, and perhaps also directly transfer the SAM system into the endogenous brain astrocytes, thus without the need of implanting foreign cells.

Double-stranded DNA breaks (DSB) occur when using CRISPR, causing mutations that could transform the cells and alter gene expression [25, 68–71]. However, the SAM system does not cause DSB and thus does not present this kind of problem. Host immune response to Cas proteins can be a concern [72], but as other studies have shown, the improvement of less immunogenic, higher fidelity CRISPR-Cas systems, together with new, more specific and safer delivery systems may overcome the aforementioned problems associated with the use of CRISPR-Cas for therapy [73–75].

The therapeutic approach used can be improved by activating the expression of neurotrophic factors such as BDNF and GDNF that can increase DA metabolism in the *striatum* [56, 76, 77], or the use pharmacological inhibitors of COMT and MAO-B to increase the levels of DA in the extracellular space.

In summary, we showed a proof-of-concept experiment that demonstrates the functional capacity of astrocytes expressing their endogenous *th* gene to produce DA and induce behavioral motor improvement in an experimental rat model of PD. This same approach can be improved by achieving the expression of other proteins that can protect the DA neurons and using different cell types like stem cells or induced pluripotent stem cells to better integrate the cells within the basal ganglia neuronal circuits.

### Supplementary information

Supplementary Information

## Data Availability

Data analyzed in this study are available from the corresponding author upon reasonable request.

## References

[1] Tysnes OB, Storstein A (2017). Epidemiology of Parkinson’s disease. J Neural Transm.

[2] Hartmann A (2004). Postmortem studies in Parkinson’s disease. Dialogues Clin Neurosci.

[3] Surmeier DJ (2017). Determinants of dopaminergic neuron loss in Parkinson’s disease. Physiol Behav.

[4] Nguyen M, Wong YC, Ysselstein D, Severino A, Krainc D (2019). Synaptic, mitochondrial, and lysosomal dysfunction in Parkinson’s disease dopaminergic neurodegeneration in PD. Trends Neurosci.

[5] Poewe W, Seppi K, Tanner CM, Halliday GM, Brundin P, Volkmann J (2017). Parkinson disease. Nat Rev Dis Primers.

[6] Goetz CG. The history of Parkinson’s disease: early clinical descriptions and neurological therapies. Cold Spring Harb Perspect Med. 2011;1:a008862. 10.1101/cshperspect.a008862 PMC3234454 22229124

[7] You H, Mariani LL, Mangone G, Le Febvre de Nailly D, Charbonnier-Beaupel F, Corvol JC (2018). Molecular basis of dopamine replacement therapy and its side effects in Parkinson’s disease. Cell Tissue Res.

[8] Bastide MF, Meissner WG, Picconi B, Fasano S, Fernagut PO, Feyder M (2015). Pathophysiology of L-dopa-induced motor and non-motor complications in Parkinson’s disease. Prog Neurobiol.

[9] Vijayakumar D, Jankovic J (2016). Drug-induced dyskinesia, part 1: treatment of levodopa-induced dyskinesia. Drugs..

[10] Schapira AHV, Chaudhuri KR, Jenner P. Non-motor features of Parkinson disease. Nat Rev Neurosci. 2017;18:435–50. 10.1038/nrn.2017.62 28592904

[11] Volkmann J, Albanese A, Antonini A, Chaudhuri KR, Clarke CE, De Bie RMA (2013). Selecting deep brain stimulation or infusion therapies in advanced Parkinson’s disease: an evidence-based review. J Neurol.

[12] Kikuchi T, Morizane A, Doi D, Magotani H, Onoe H, Hayashi T (2017). Human iPS cell-derived dopaminergic neurons function in a primate Parkinson’s disease model. Nature..

[13] Axelsen TM, Woldbye DPD (2018). Gene therapy for Parkinson’s disease, an update. J Parkinsons Dis.

[14] Luo J, Kaplitt MG, Fitzsimons HL, Zuzga DS, Liu Y, Oshinsky ML (2002). Subthalamic GAD gene therapy in a Parkinson’s disease rat model. Science..

[15] Kaplitt MG, Feigin A, Tang C, Fitzsimons HL, Mattis P, Lawlor PA (2007). Safety and tolerability of gene therapy with an adeno-associated virus (AAV) borne GAD gene for Parkinson’s disease: an open label, phase I trial. Lancet..

[16] Campos-Romo A, Ojeda-Flores R, Moreno-Briseño P, Vergara P, Segovia J, Carrillo-Ruiz JD (2012). Behavioral improvement in MPTP-treated nonhuman primates in the HALLWAY task after transfer of TH cDNA to host astrocytes. Acta Neurobiol Exp.

[17] Goodwin LO, Splinter E, Davis TL, Urban R, He H, Braun RE (2019). Large-scale discovery of mouse transgenic integration sites reveals frequent structural variation and insertional mutagenesis. Genome Res.

[18] Muhuri M, Maeda Y, Ma H, Ram S, Fitzgerald KA, Tai PWL, et al. Overcoming innate immune barriers that impede AAV gene therapy vectors. J Clin Invest. 2021;131:e143780. 10.1172/JCI143780 PMC7773343 33393506

[19] Hartmann A (2004). Postmortem studies in Parkinson’s disease: the role of human postmortem studies in PD research. LLS SAS. Dialog Clin Neurosci.

[20] Tansey MG, Romero-Ramos M (2019). Immune system responses in Parkinson’s disease: early and dynamic. Eur J Neurosci.

[21] Asanuma M, Miyazaki I, Murakami S, Diaz-Corrales FJ, Ogawa N. Striatal astrocytes act as a reservoir for L-DOPA. PLoS One. 2014;9:e106362. 10.1371/journal.pone.0106362 PMC4154692 25188235

[22] Juorio AV, Li XM, Walz W, Paterson IA (1993). Decarboxylation of l-Dopa by cultured mouse astrocytes. Brain Res.

[23] Segovia J, Vergara P, Brenner M (1998). Astrocyte-specific expression of tyrosine hydroxylase after intracerebral gene transfer induces behavioral recovery in experimental Parkinsonism. Gene Ther.

[24] Cortez N, Trejo F, Vergara P, Segovia J (2000). Primary astrocytes retrovirally transduced with a tyrosine hydroxylase transgene driven by a Glial-Specific promoter elicit behavioral recovery in experimental Parkinsonism. J Neurosci Res.

[25] Hsu PD, Scott DA, Weinstein JA, Ran FA, Konermann S, Agarwala V (2013). DNA targeting specificity of RNA-guided Cas9 nucleases. Nat Biotechnol.

[26] Ran FA, Hsu PD, Wright J, Agarwala V, Scott DA, Zhang F (2013). Genome engineering using the CRISPR-Cas9 system. Nat Protoc.

[27] Konermann S, Brigham MD, Trevino AE, Joung J, Abudayyeh OO, Barcena C (2015). Genome-scale transcriptional activation by an engineered CRISPR-Cas9 complex. Nature..

[28] Chavez A, Tuttle M, Pruitt BW, Ewen-Campen B, Chari R, Ter-Ovanesyan D (2016). Comparison of Cas9 activators in multiple species. Nat Methods.

[29] Zhang Y, Yin C, Zhang T, Li F, Yang W, Kaminski R (2015). CRISPR/gRNA-directed synergistic activation mediator (SAM) induces specific, persistent and robust reactivation of the HIV-1 latent reservoirs. Sci Rep.

[30] National Research Council (U.S.) Committee for the Update of the Guide for the Care and Use of Laboratory Animals., Institute for Laboratory Animal Research (U.S.). Guide for the care and use of laboratory animals. 8th ed. Washington (DC): National Academies Press (US); 2011. 220.

[31] Erlij D, Acosta-García J, Rojas-Márquez M, González-Hernández B, Escartín-Perez E, Aceves J (2012). Dopamine D4 receptor stimulation in GABAergic projections of the globus pallidus to the reticular thalamic nucleus and the substantia nigra reticulata of the rat decreases locomotor activity. Neuropharmacology..

[32] Gu MJ, Jeon JH, Oh MS, Hong SP (2016). Measuring levels of biogenic amines and their metabolites in rat brain tissue using high-performance liquid chromatography with photodiode array detection. Arch Pharm Res.

[33] Cruz-Trujillo R, Avalos-Fuentes A, Rangel-Barajas C, Paz-Bermúdez F, Sierra A, Escartín-Perez E (2013). D3 dopamine receptors interact with dopamine D1 but not D4 receptors in the GABAergic terminals of the SNr of the rat. Neuropharmacology..

[34] Guerrero-Cázares H, del Alatorre-Carranza MP, Delgado-Rizo V, Duenas-Jimenez JM, Mendoza-Magana ML (2007). Dopamine release modifies intracellular calcium levels in tyrosine hydroxylase-transfected C6 cells. Brain Res Bull.

[35] Paxinos G, Watson C, The rat brain, in stereotaxic coordinates. San Diego: Academic Press; 1997.

[36] Hudson JL, van Horne CG, Strömberg I, Brock S, Clayton J, Masserano J (1993). Correlation of apomorphine- and amphetamine-induced turning with nigrostriatal dopamine content in unilateral 6-hydroxydopamine lesioned rats. Brain Res.

[37] Song JJ, Oh SM, Kwon OC, Wulansari N, Lee HS, Chang MY (2018). Cografting astrocytes improves cell therapeutic outcomes in a Parkinson’s disease model. J Clin Investig.

[38] Björklund A, Dunnett SB (2019). The amphetamine induced rotation test: a re-assessment of its use as a tool to monitor motor impairment and functional recovery in rodent models of Parkinson’s disease. J Parkinsons Dis.

[39] Su RJ, Zhen JI, Wang W, Zhang JL, Zheng Y, Wang XM (2018). Time - course behavioral features are correlated with Parkinson’s disease - associated pathology in a 6 - hydroxydopamine hemiparkinsonian rat model. Mol Med Rep.

[40] Landers MR, Kinney JW, Van Breukelen F (2014). Forced exercise before or after induction of 6-OHDA-mediated nigrostriatal insult does not mitigate behavioral asymmetry in a hemiparkinsonian rat model. Brain Res.

[41] Drucker-Colín R, García-Hernández F (1991). A new motor test sensitive to aging and dopaminergic function. J Neurosci Methods.

[42] Paxinos G, Watson C. The rat brain in stereotaxic coordinates. London: Academic Press; 2007.

[43] Iyer V, Venkiteswaran K, Savaliya S, Lieu CA, Handly E, Gilmour TP, et al. The cross-hemispheric nigrostriatal pathway prevents the expression of levodopa-induced dyskinesias. Neurobiol Dis. 2021;159:105491. 10.1016/j.nbd.2021.105491 PMC8597404 34461264

[44] Schlachetzki JCM, Marxreiter F, Regensburger M, Kulinich A, Winner B, Winkler J (2014). Increased tyrosine hydroxylase expression accompanied by glial changes within the non-lesioned hemisphere in the 6-hydroxydopamine model of Parkinson’s disease. Restor Neurol Neurosci.

[45] Gándara C, Affleck V, Stoll EA (2018). Manufacture of third-generation lentivirus for preclinical use, with process development considerations for translation to good manufacturing practice. Hum Gene Ther Methods.

[46] Zufferey R, Dull T, Mandel RJ, Bukovsky A, Quiroz D, Naldini L (1998). Self-inactivating lentivirus vector for safe and efficient in vivo gene delivery. J Virol.

[47] Gundlach AL, Beart PM (1982). Neurochemical studies of the mesolimbic dopaminergic pathway: glycinergic mechanisms and glycinergic‐dopaminergic interactions in the rat ventral tegmentum. J Neurochem.

[48] Carder RK, Jackson D, Morris HJ, Lund RD, Zigmond MJ (1989). Dopamine released from mesencephalic transplants restores modulation of striatal acetylcholine release after neonatal 6-hydroxydopamine: an in vitro analysis. Exp Neurol.

[49] Cragg SJ, Clarke DJ, Greenfield SA (2000). Real-time dynamics of dopamine released from neuronal transplants in experimental Parkinson’s disease. Exp Neurol.

[50] Schwarting RKW, Huston JP. The unilateral 6-hydroxydopamine lesion model in behavioral brain research. Analysis of functional deficits, recovery and treatments. Progress Neurobiol. 1996;50:275–331. 10.1016/s0301-0082(96)00040-8 8971983

[51] Ungerstedt U (1971). Postsynaptic supersensitivity after 6‐hydroxy‐dopamine induced degeneration of the nigro‐striatal dopamine system. Acta Physiol Scand.

[52] Sauer H, Oertel WH. Progressive degeneration of nigrostriatal dopamine neurons following intrastriatal terminal lesions with 6-hydroxydopamine: a combined retrograde tracing and immunocytochemical study in the rat. Neuroscience. 1994;59:401–15. 10.1016/0306-4522(94)90605-x 7516500

[53] Zuch CL, Nordstroem VK, Briedrick LA, Hoernig GR, Granholm AC, Bickford PC (2000). Time course of degenerative alterations in nigral dopaminergic neurons following a 6-hydroxydopamine lesion. J Comp Neurol.

[54] Labandeira-Garcia JL, Rozas G, Lopez-Martin E, Liste I, Guerra MJ (1996). Time course of striatal changes induced by 6-hydroxydopamine lesion of the nigrostriatal pathway, as studied by combined evaluation of rotational behaviour and striatal Fos expression. Exp Brain Res.

[55] Kirik D, Cederfjäll E, Halliday G, Petersén A. Gene therapy for Parkinson’s disease: disease modification by GDNF family of ligands. Neurobiol Dis. 2017;97:179–88. 10.1016/j.nbd.2016.09.008 27616425

[56] Allen SJ, Watson JJ, Shoemark DK, Barua NU, Patel NK (2013). GDNF, NGF and BDNF as therapeutic options for neurodegeneration. Pharmacol Therapeutics.

[57] Choi DJ, Kwon JK, Joe EH (2018). A Parkinson’s disease gene, DJ-1, regulates astrogliosis through STAT3. Neurosci Lett.

[58] Batassini C, Broetto N, Tortorelli LS, Borsoi M, Zanotto C, Galland F, et al. Striatal injury with 6-OHDA transiently increases cerebrospinal GFAP and S100B. Neural Plast. 2015;2015:387028. 10.1155/2015/387028 PMC4451977 26090233

[59] Nomura T, Yabe T, Rosenthal ES, Krzan M, Schwartz JP (2000). PSA-NCAM distinguishes reactive astrocytes in 6-OHDA-lesioned substantia nigra from those in the striatal terminal fields. J Neurosci Res.

[60] Horellou P, Marlier L, Privat A, Mallet J (1990). Behavioural effect of engineered cells that synthesize L‐DOPA or dopamine after grafting into the rat neostriatum. Eur J Neurosci.

[61] Truong L, Allbutt H, Kassiou M, Henderson JM (2006). Developing a preclinical model of Parkinson’s disease: a study of behaviour in rats with graded 6-OHDA lesions. Behav Brain Res.

[62] Torres EM, Dunnett SB (2007). Amphetamine induced rotation in the assessment of lesions and grafts in the unilateral rat model of Parkinson’s disease. Eur Neuropsychopharmacol.

[63] Abrous DN, Shaltot ARA, Torres EM, Dunnett SB (1993). Dopamine-rich grafts in the neostriatum and/or nucleus accumbens: effects on drug-induced behaviours and skilled paw-reaching. Neuroscience..

[64] Corkrum M, Covelo A, Lines J, Bellocchio L, Pisansky M, Loke K (2020). Dopamine-evoked synaptic regulation in the nucleus accumbens requires astrocyte activity. Neuron..

[65] Zhang Y, Yin C, Zhang T, Li F, Yang W, Kaminski R, et al. CRISPR/gRNA-directed synergistic activation mediator (SAM) induces specific, persistent and robust reactivation of the HIV-1 latent reservoirs. Sci Rep. 2015;5;16277. 10.1038/srep16277 PMC4633726 26538064

[66] Xiong K, Zhou Y, Hyttel P, Bolund L, Freude KK, Luo Y (2016). Generation of induced pluripotent stem cells (iPSCs) stably expressing CRISPR-based synergistic activation mediator (SAM). Stem Cell Res.

[67] Wang CH, Lundh M, Fu A, Kriszt R, Huang TL, Lynes MD, et al. CRISPR-engineered human brown-like adipocytes prevent diet-induced obesity and ameliorate metabolic syndrome in mice. Sci Transl Med. 2020;12:eaaz8664. 10.1126/scitranslmed.aaz8664 PMC7704293 32848096

[68] Fu Y, Foden JA, Khayter C, Maeder ML, Reyon D, Joung JK (2013). High-frequency off-target mutagenesis induced by CRISPR-Cas nucleases in human cells. Nat Biotechnol.

[69] Leibowitz ML, Papathanasiou S, Doerfler PA, Blaine LJ, Sun L, Yao Y (2021). Chromothripsis as an on-target consequence of CRISPR–Cas9 genome editing. Nat Genet.

[70] Nahmad AD, Reuveni E, Goldschmidt E, Tenne T, Liberman M, Horovitz-Fried M, et al. Frequent aneuploidy in primary human T cells after CRISPR–Cas9 cleavage. Nat Biotechnol. 2022;40:1807–13. 10.1038/s41587-022-01377-0 PMC7613940 35773341

[71] Zuccaro MV, Xu J, Mitchell C, Marin D, Zimmerman R, Rana B (2020). Allele-specific chromosome removal after Cas9 cleavage in human embryos. Cell..

[72] Tang XZE, Tan SX, Hoon S, Yeo GW (2022). Pre-existing adaptive immunity to the RNA-editing enzyme Cas13d in humans. Nat Med.

[73] Bravo JPK, Liu M, sen, Hibshman GN, Dangerfield TL, Jung K, McCool RS (2022). Structural basis for mismatch surveillance by CRISPR–Cas9. Nature..

[74] Chen JS, Dagdas YS, Kleinstiver BP, Welch MM, Sousa AA, Harrington LB (2017). Enhanced proofreading governs CRISPR-Cas9 targeting accuracy. Nature..

[75] Kleinstiver BP, Pattanayak V, Prew MS, Tsai SQ, Nguyen NT, Zheng Z (2016). High-fidelity CRISPR-Cas9 nucleases with no detectable genome-wide off-target effects. Nature..

[76] Grondin R, Gash DM (1998). Glial cell line-derived neurotrophic factor (GDNF): a drug candidate for the treatment of Parkinson’s disease. J Neurol..

[77] Jin W. Regulation of BDNF‐TrkB signaling and potential therapeutic strategies for parkinson’s disease. J Clin Med. 2020;9:257. 10.3390/jcm9010257 PMC7019526 31963575

