# Supplementary material for: CRISPR/sgRNA-directed synergistic activation mediator (SAM) as a therapeutic tool for Parkinson´s disease
Source: Gene Ther. 2023 Aug 4;31(1-2):31–44. doi: 10.1038/s41434-023-00414-0 (PMC10788271; doi:10.1038/s41434-023-00414-0)
Supplement: Supplementary file 1 — Supplementary Information [file 41434_2023_414_MOESM1_ESM.pdf]

| GEN | ID | Sense | Species | FW | RW |
|---|---|---|---|---|---|
| TH | 25085 | Neg | Rat | CACCGGGTCACTTACTGTTAGACCT | AAACAGGTCTAACAGTAAGTGACCC |
| TH | 25085 | Neg | Rat | CACCGGAAGGCCTTAGGGAGCTGCC | AAACGGCAGCTCCCTAAGGCCTTCC |
| TH | 25085 | Pos | Rat | CACCGGAGACACAGAATGTTCCAGA | AAACTCTGGAACATTCTGTGTCTCC |
| TH | 25085 | Neg | Rat | CACCGGTGCCAGCACATATACCGAC | AAACGTCGGTATATGTGCTGGCACC |
| TH | 25085 | Neg | Rat | CACCGGAACATGGCCCATGTCCTGG | AAACCCAGGACATGGGCCATGTTCC |
| TH | 25085 | Pos | Rat | CACCGAGAGCTCTCTAACCAAACCA | AAACTGGTTTGGTTAGAGAGCTCTC |
| TH | 25085 | Neg | Rat | CACCGTACGTCGTGCCTCGGGCTGA | AAACTCAGCCCGAGGCACGACGTAC |
| TH | 25085 | Neg | Rat | CACCGAATTAGATCTAATGGGACGG | AAACCCGTCCCATTAGATCTAATTC |
| TH | 25085 | Pos | Rat | CACCGGCTGTGCCTGAGGGAGGCGG | AAACCCGCCTCCCTCAGGCACAGCC |
| TH | 25085 | Neg | Rat | CACCGGCCCCCTCGCCACAGCCCAA | AAACTTGGGCTGTGGCGAGGGGGCC |
| TH | 25085 | Neg | Rat | CACCGGCAGGATGCCAAGCAGGCCG | AAACCGGCCTGCTTGGCATCCTGCC |
| TH | 25085 | Neg | Rat | CACCGAGCCCTTAGGAAATCCAGCA | AAACTGCTGGATTTCCTAAGGGCTC |
| TH | 25085 | Neg | Rat | CACCGCCTGCCCTACGTCGTGCCTC | AAACGAGGCACGACGTAGGGCAGGC |

Supplementary Table 1. Sequences tested as *Th sgRNA*.

Supplementary Figure 1.

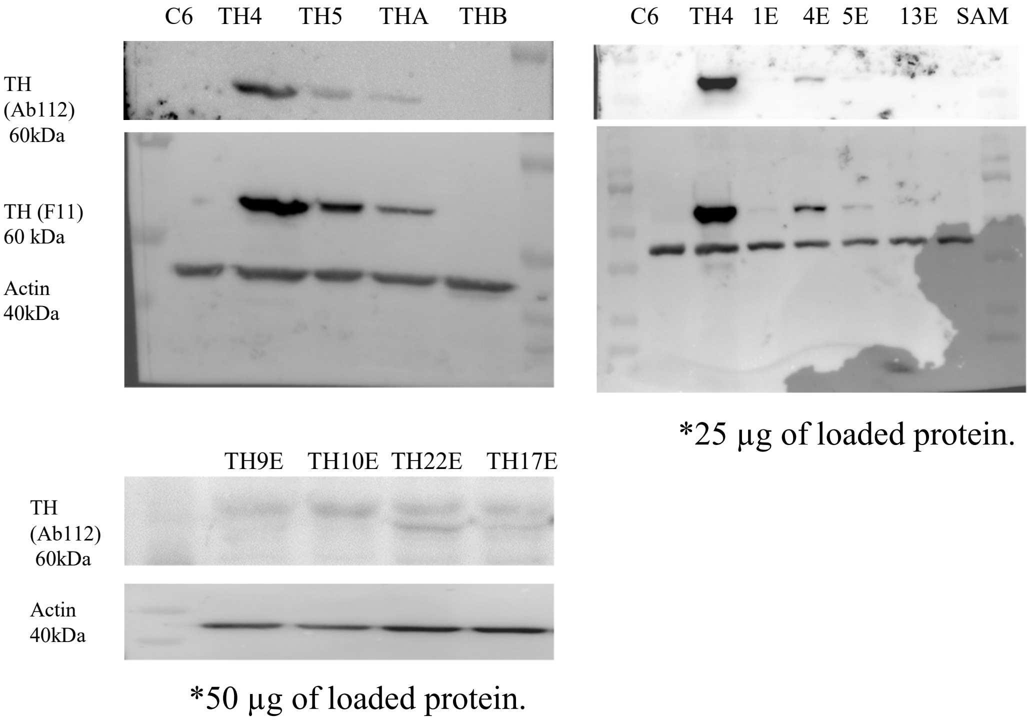


A) Western Blot of the tested *Th* sgRNA in the transfected C6 cell line.

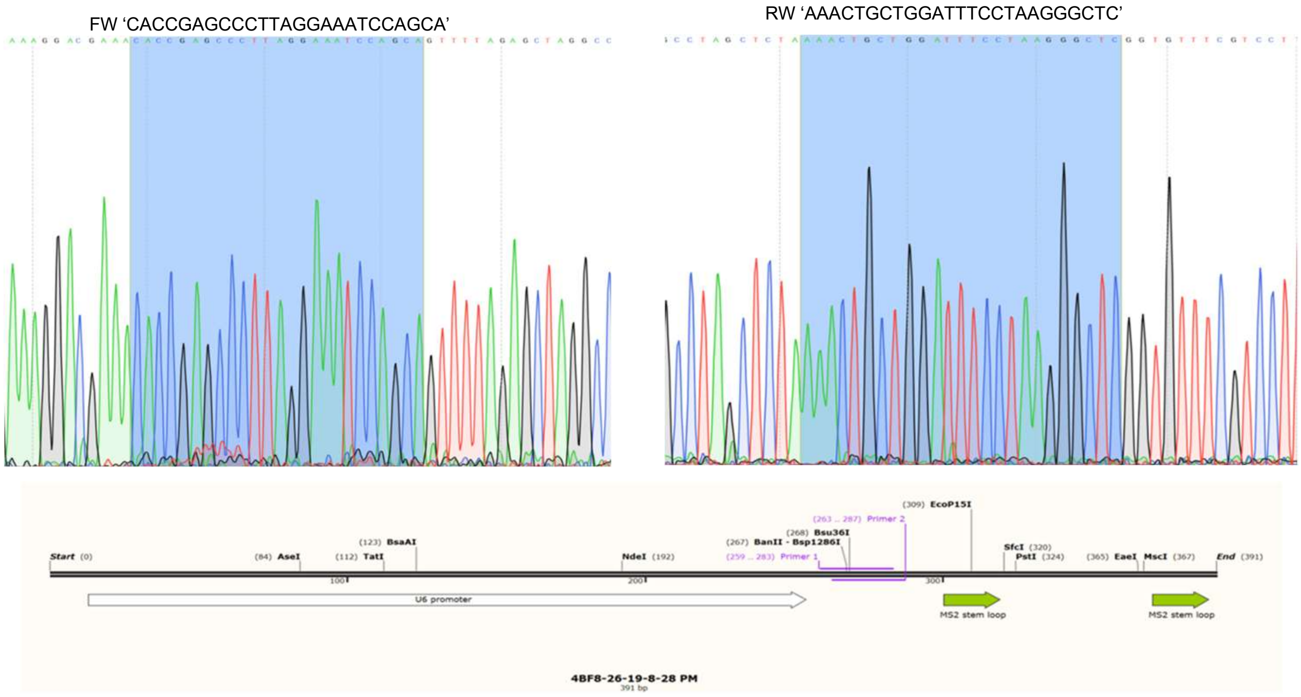


B) Sequencing *Th*4 sgRNA

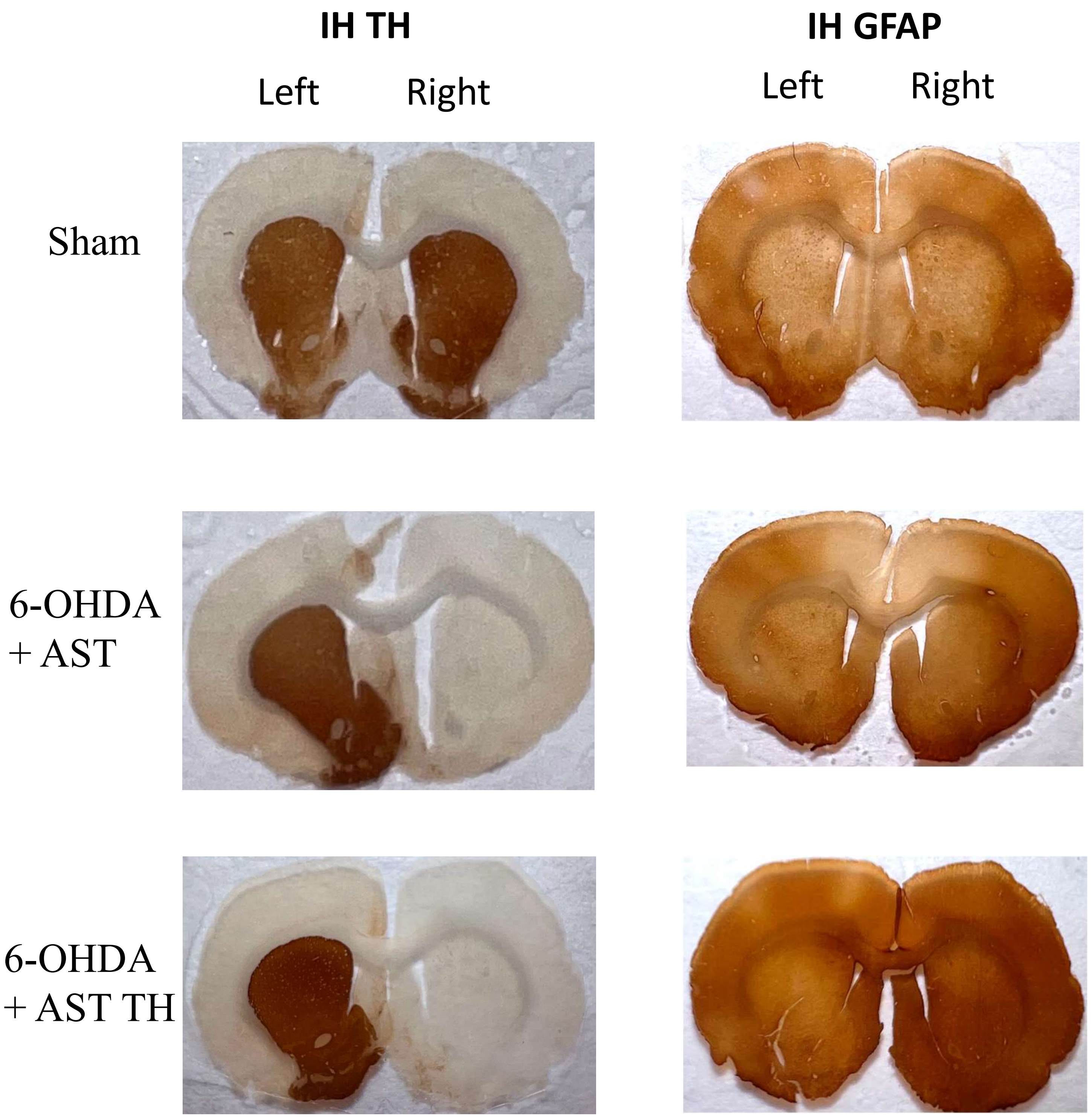


Supplementary Figure 2. TH and GFAP Immunochemistry brain slices of the experimental groups.

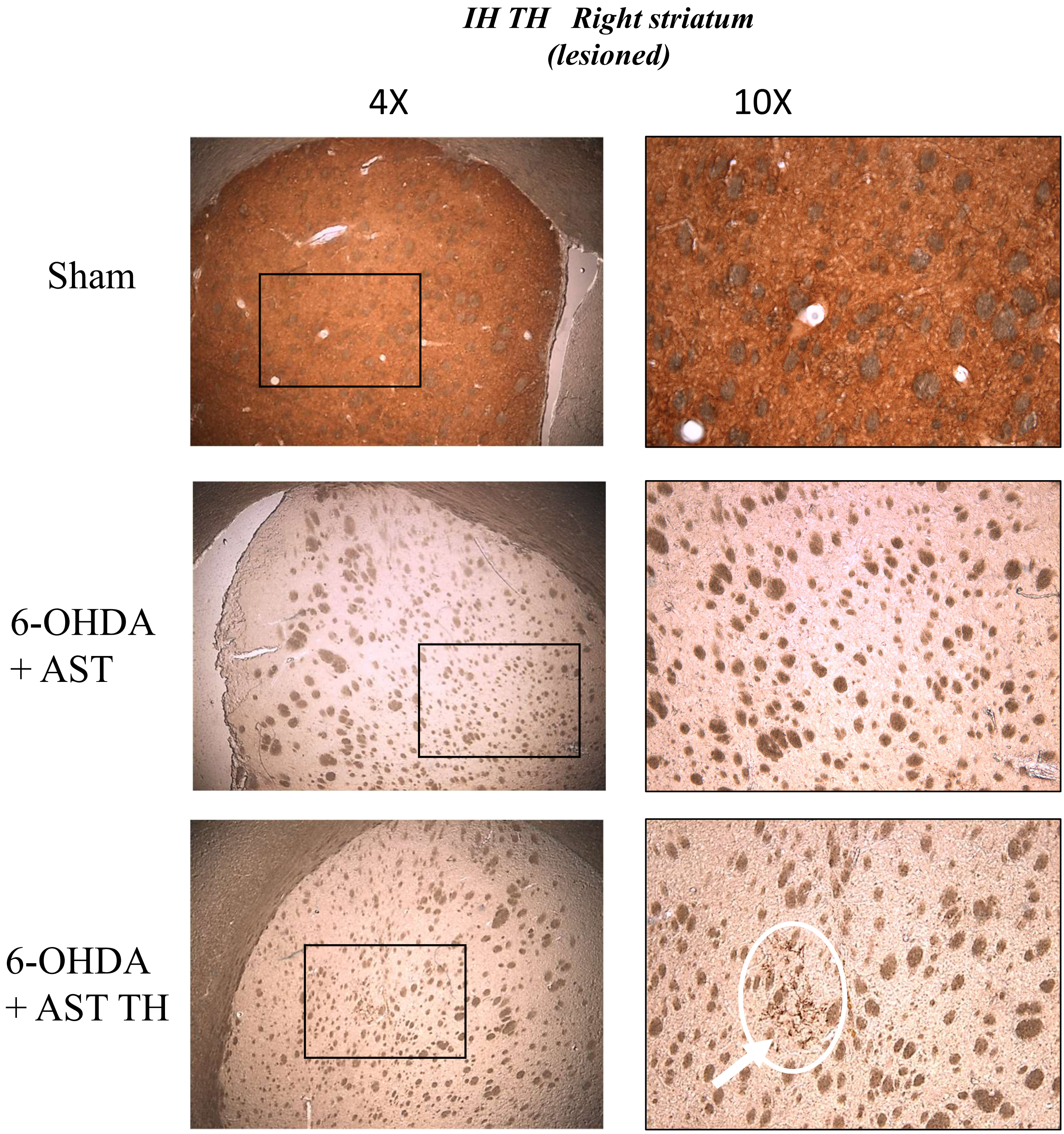


Supplementary Figure 3A. TH Immunohistochemistry of 6-OHDA lesioned (right) *striatum* side between the experimental groups.

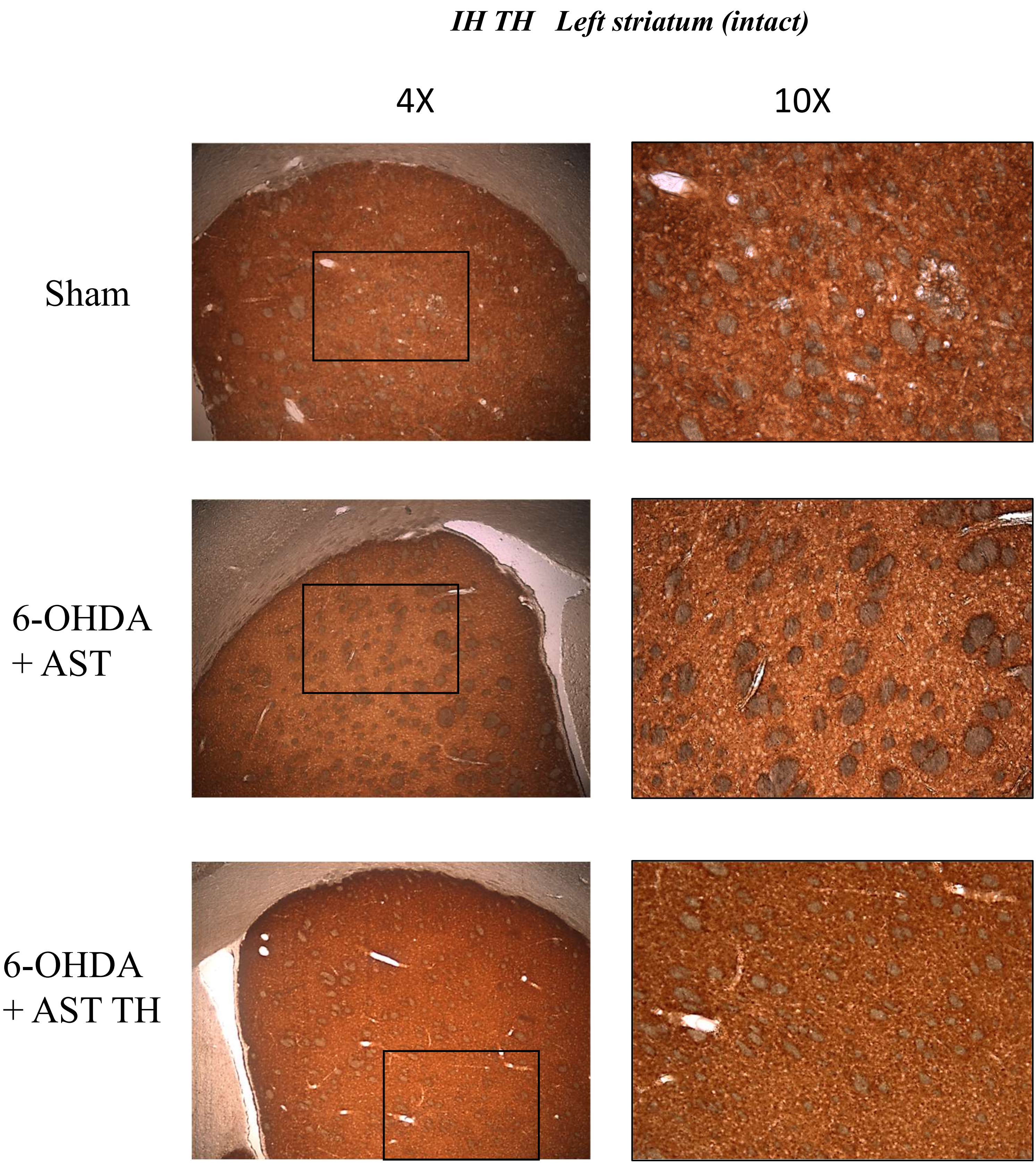


Supplementary Figure 3B. TH Immunohistochemistry of intact (left) *striatum* side between the experimental groups.

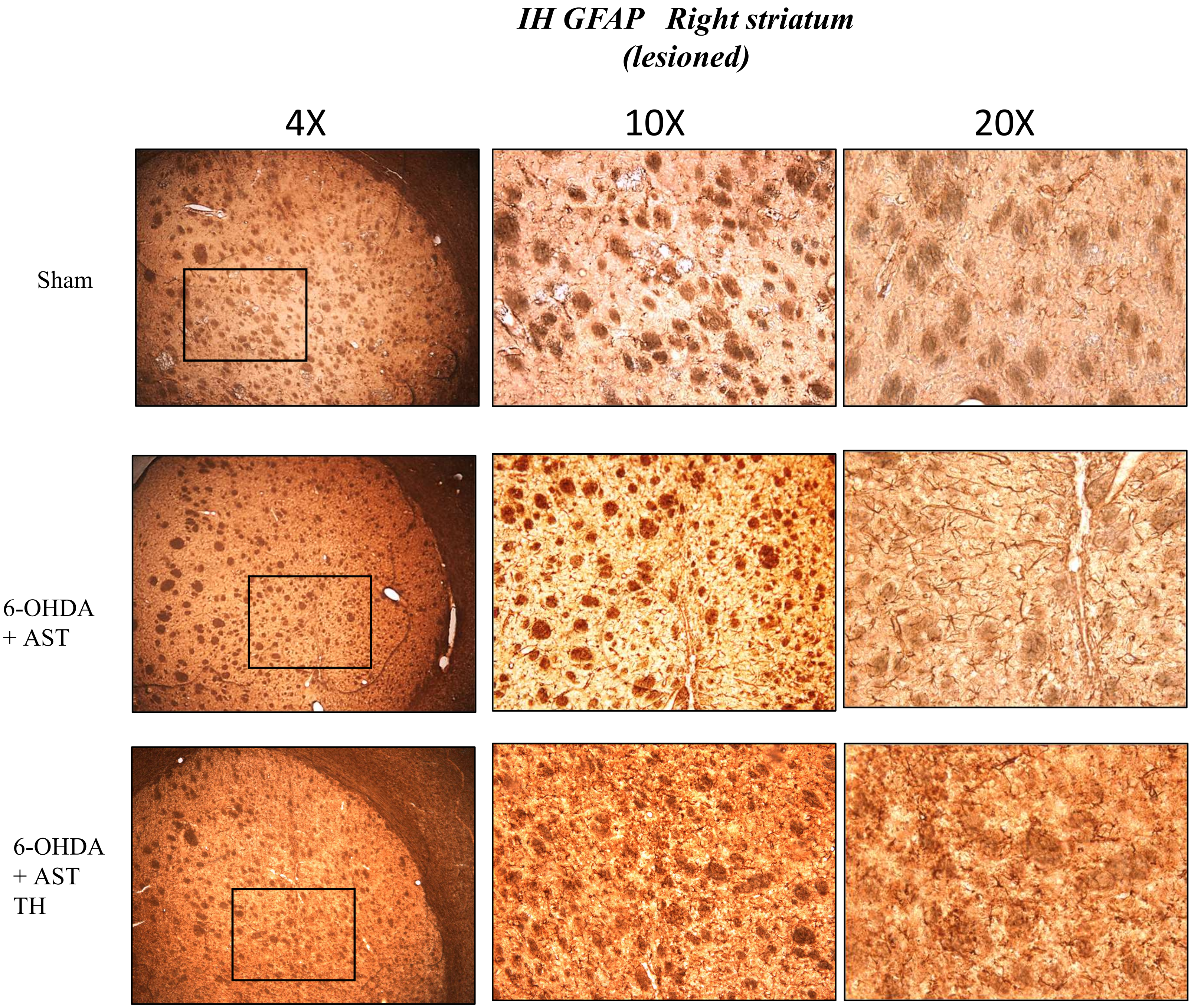


Supplementary Figure 4A. GFAP Immunohistochemistry of 6-OHDA lesioned (right) *striatum* side between the experimental groups.

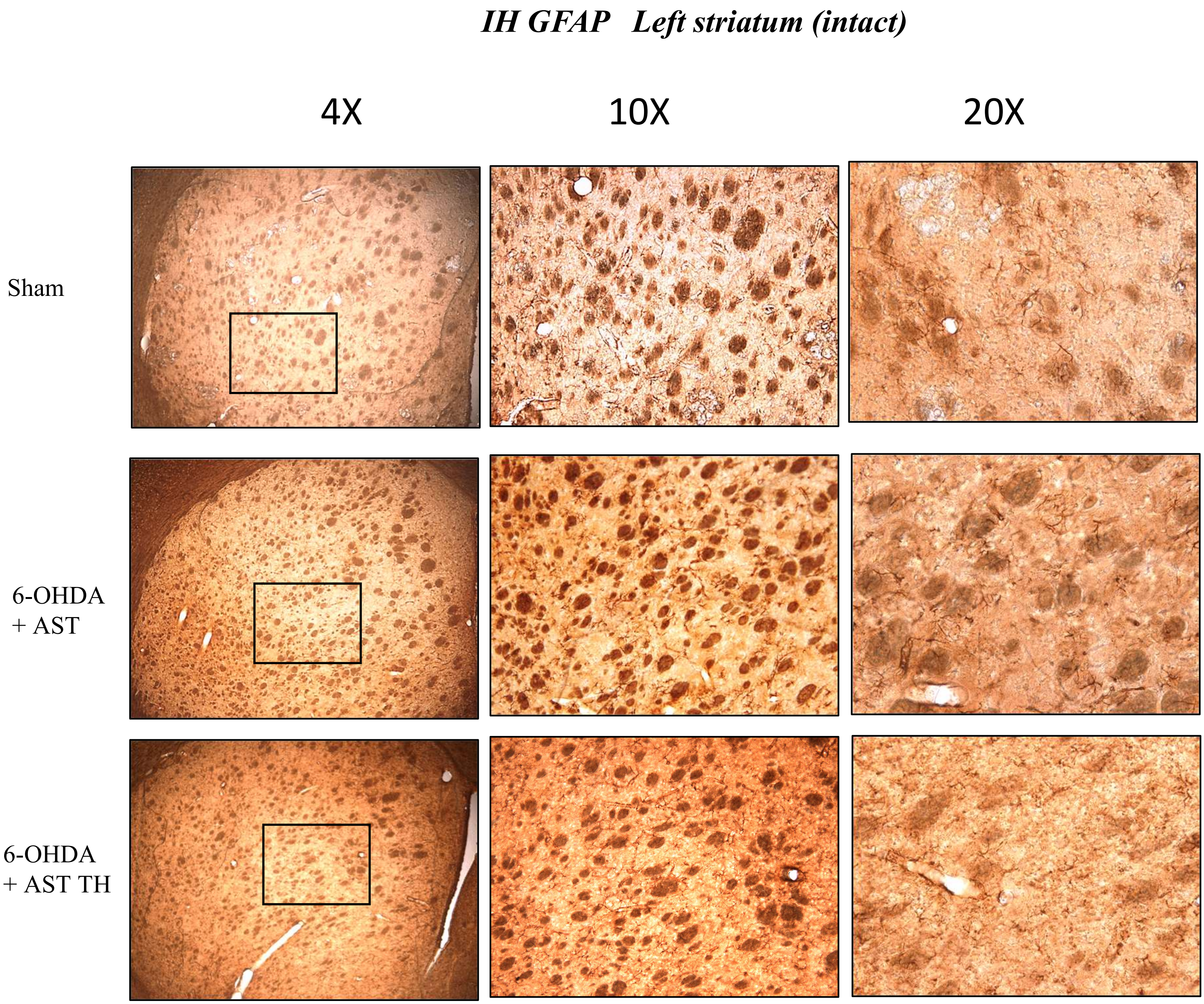


Supplementary Figure 4B. GFAP Immunohistochemistry of intact (left) *striatum* side between the experimental groups.

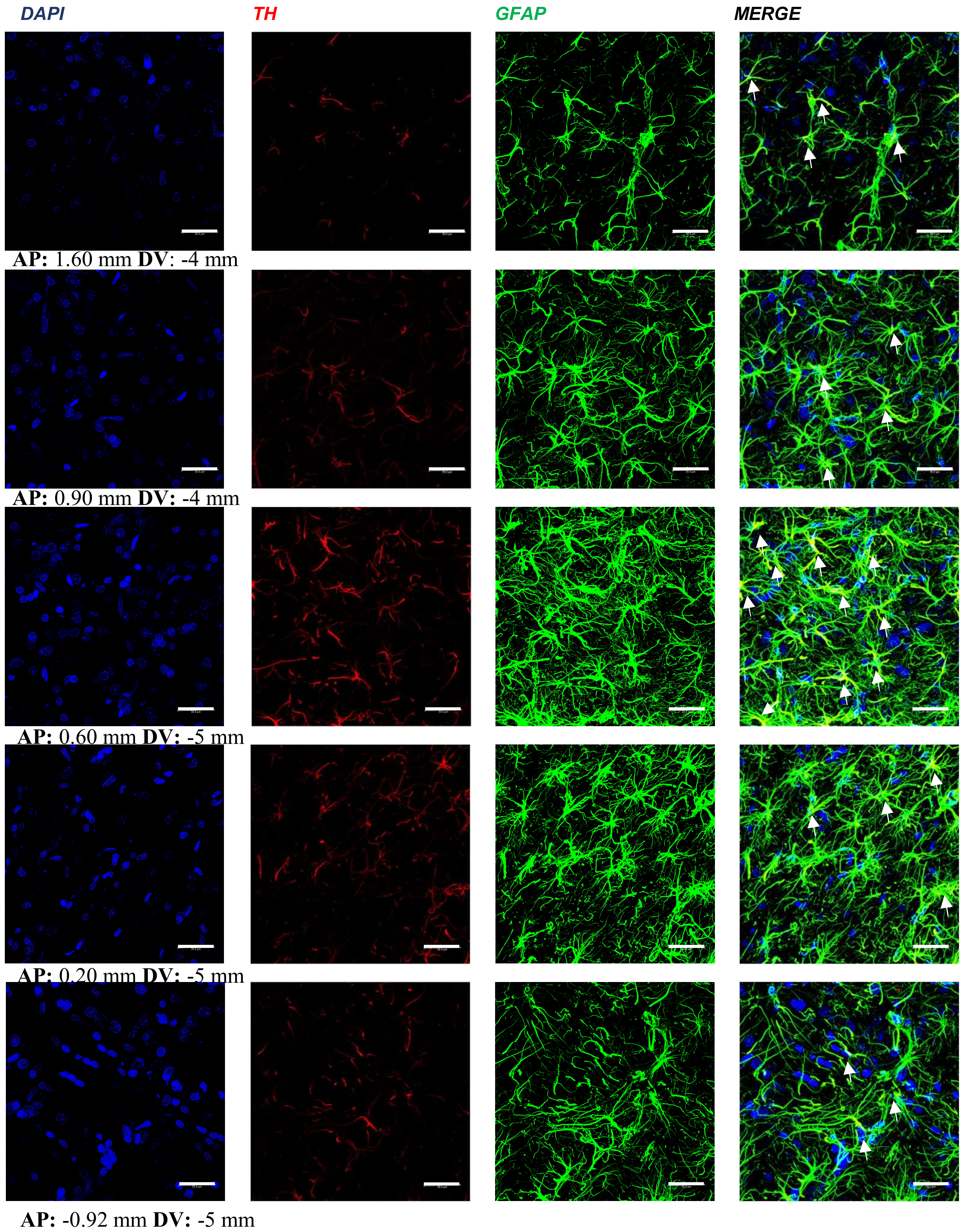


Supplementary Figure 5. Complementary figure of confocal images presents at Figure 6. DAPI (nuclei) in blue fluorescent signal, Th is revealed with red, and GFAP with a green fluorescent signal. Cells expressing both Th and GFAP show a yellow signal. Bars represent 30 µm.

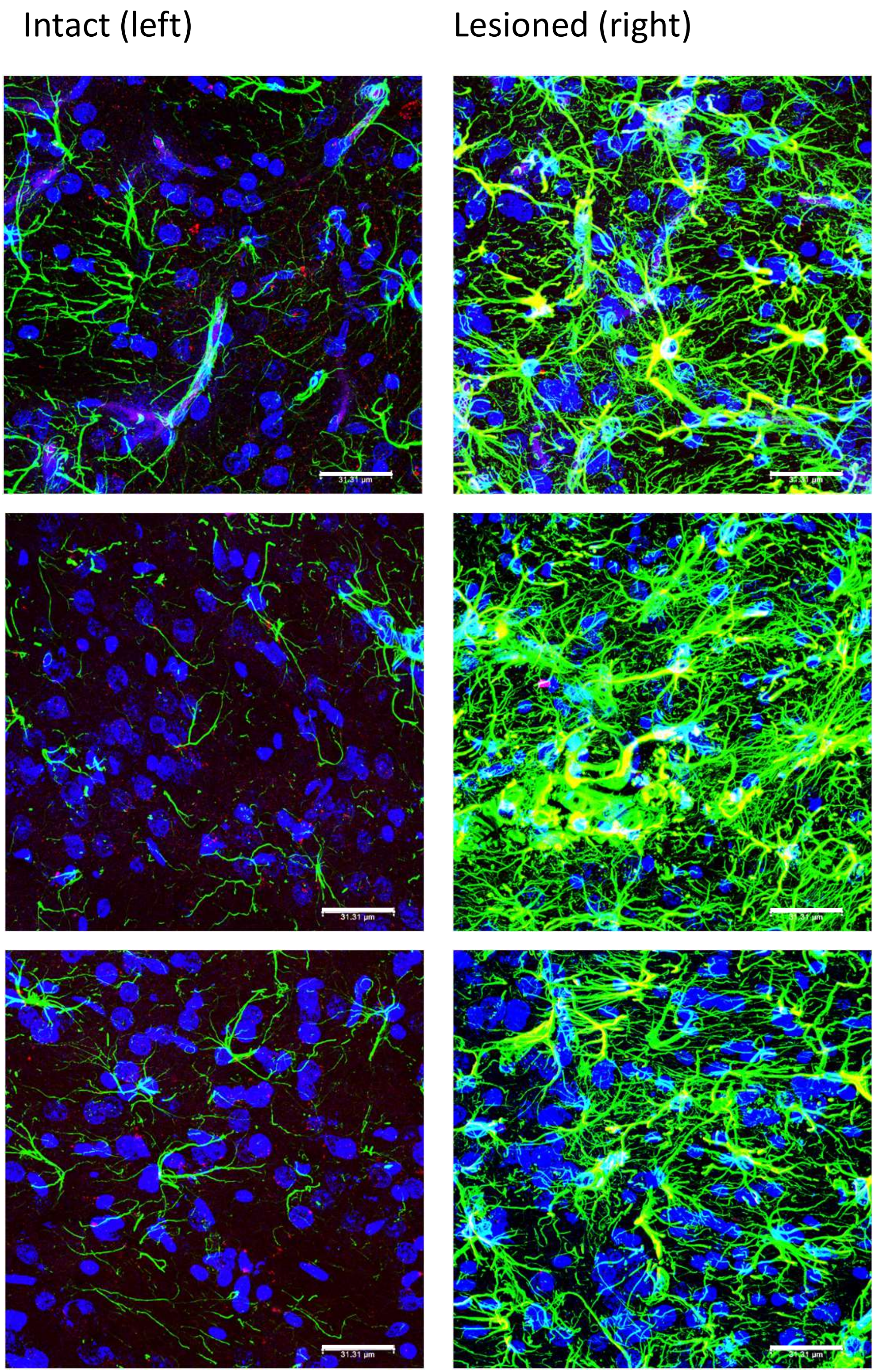


Supplementary Figure 6. GFAP expression between intact and 6-OHDA lesioned *striatum* side of 6-OHDA + AST TH group. DAPI (nuclei) in blue fluorescent signal, Th is revealed with red, and GFAP with a green fluorescent signal. Bars represent 30 µm.
